# Increased branching independent of strigolactone in cytokinin oxidase 2-overexpressing tomato is mediated by reduced auxin transport

**DOI:** 10.1186/s43897-022-00032-1

**Published:** 2022-05-03

**Authors:** Lilian Ellen Pino, Joni E. Lima, Mateus H. Vicente, Ariadne F. L. de Sá, Francisco Pérez-Alfocea, Alfonso Albacete, Juliana L. Costa, Tomáš Werner, Thomas Schmülling, Luciano Freschi, Antonio Figueira, Agustin Zsögön, Lázaro E. P. Peres

**Affiliations:** 1 Laboratory of Plant Breeding, Centro de Energia Nuclear na Agricultura, University of Sao Paulo, São Paulo, Brazil; 2 Laboratory of Hormonal Control of Plant Development, Department of Biological Sciences, Escola Superior de Agricultura ‘Luiz de Queiroz’University of Sao Paulo, Piracicaba, Brazil; 3 Botany Department, ICB, Federal University of Minas Gerais, Belo Horizonte, Brazil; 4 Department of Plant Nutrition, CEBAS-CSIC, Campus Univ. Espinardo, Murcia, Spain; 5 Institute of Biology/Applied Genetics, Freie Universität Berlin, Berlin, Germany; 6 Institute of Biology, University of Graz, Schubertstraße 51, 8010 Graz, Austria; 7 Biosciences Institute, University of Sao Paulo, São Paulo, Brazil; 8 Plant Sciences Department, Federal University of Viçosa, Viçosa, Brazil

**Keywords:** CKX2, Hormones, Plant development, *Solanum*

## Abstract

Tomato production is influenced by shoot branching, which is controlled by different hormones. Here we produced tomato plants overexpressing the cytokinin-deactivating gene *CYTOKININ OXYDASE 2* (*CKX2*). *CKX2*-overexpressing (CKX2-OE) plants showed an excessive growth of axillary shoots, the opposite phenotype expected for plants with reduced cytokinin content, as evidenced by LC-MS analysis and *ARR5-GUS* staining. The TCP transcription factor *SlBRC1b* was downregulated in the axillary buds of CKX2-OE and its excessive branching was dependent on a functional version of the GRAS-family gene *LATERAL SUPPRESSOR* (*LS*). Grafting experiments indicated that increased branching in CKX2-OE plants is unlikely to be mediated by root-derived signals. Crossing CKX2-OE plants with transgenic antisense plants for the strigolactone biosynthesis gene *CAROTENOID CLEAVAGE DIOXYGENASE* (*CCD7*-AS) produced an additive phenotype, indicating independent effects of cytokinin and strigolactones on increased branching. On the other hand, CKX2-OE plants showed reduced polar auxin transport and their bud outgrowth was reduced when combined with auxin mutants. Accordingly, CKX2-OE basal buds did not respond to auxin applied in the decapitated apex. Our results suggest that tomato shoot branching depends on a fine-tuning of different hormonal balances and that perturbations in the auxin status could compensate for the reduced cytokinin levels in CKX2-OE plants.

**Supplementary Information:**

The online version contains supplementary material available at 10.1186/s43897-022-00032-1.

## Core

Tomato plants overexpressing the cytokinin-deactivating gene *CYTOKININ OXYDASE 2* (*CKX2*) showed an excessive growth of axillary shoots, which is the opposite phenotype to that expected for plants with reduced cytokinin content. Here we provide evidence that such phenotype can be explained by disturbances in auxin status, as well as in the expression of genes associated with branching and cytokinin homeostasis.

## Gene & accession numbers

*DIAGEOTROPICA* (*DGT*), Solyc01g111170; *ENTIRE* (*E*), Solyc04g076850; *LATERAL SUPPRESSOR* (*LS*), Solyc07g066250; *SlCCD7*, Solyc01g090660. A list of genes and sequences used in the qRT-PCR analysis can be found in Supplementary Table S2.

## Introduction

Side branching is an almost universal feature of vascular plants, with important consequences for agriculture. In many horticultural crops, including tomato (*Solanum lycopersicum L*.), side branching is undesirable and incurs considerable costs in labour and management (Ward and Leyser, 2004). Thus, elimination or, at least, reduction of side branching is a long-sought goal in breeding as it would eliminate the need for repeated pruning operations (Zsögön and Peres, 2019). The *LATERAL SUPPRESSOR* (*LS*) (Schumacher et al., 1999) and *BLIND* (*BL*) (Schmitz et al., 2002) genes of tomato encode GRAS and MYB-domain transcription factors whose loss of function leads to suppressed side branching. However, exploitation of these mutants in tomato breeding has been hampered by their large pleiotropic effects on plant development that negatively impact fruit yield and quality (Groot et al., 1994). A deeper understanding of the molecular mechanisms that govern bud formation and outgrowth is needed before biotechnological manipulation of side branching can be deployed.

Side branches can form from the main axis by either subdivision of the apical meristem or, more commonly, from axillary meristems (AMs) located in the leaf axils. AMs usually remain dormant until activated by the proper endogenous or environmental cues, such as, for instance, the plant entering the flowering stage or irradiance and nutrient availability (Beveridge et al., 2003; de Jong et al., 2019). The ontogenetic origin of AMs is still unclear as evidence exists for either a few pluripotent from the apical meristem being ‘left behind’ and forming a new meristematic niche (the “detached meristem model”) or in loco de-differentiation of cells in leaf axil to form a new meristematic niche as the leaf develops (the “*de novo* induction model”) (Shi et al., 2016). Either way, side branching occurs in two successive steps: 1- initiation of AMs and 2- outgrowth, with an intervening dormancy period of variable duration (Wang and Jiao, 2018).

The phytohormone auxin is well-known to play an important role in inhibiting the outgrowth of axillary buds, a phenomenon known as apical dominance (Cline, 1994; Ferguson and Beveridge, 2009; Ward and Leyser, 2004; Barbier et al., 2018). However, the role of auxin in the process of axillary meristem formation is largely unknown. Disruption of polar auxin transport compromises auxin depletion from the leaf axil and axillary meristem initiation. Ectopic auxin biosynthesis in leaf axils interferes with axillary meristem formation, whereas repression of auxin signaling in polar auxin transport mutants can largely rescue their branching defects. These evidences strongly suggest that auxin depletion from leaf axils is a prerequisite for axillary meristem formation during vegetative development (Wang et al., 2014a,b). Lateral bud outgrowth is inhibited by the auxin derived from the shoot apex (‘apical dominance’) that moves basipetally down the stem, as proven repeatedly by elegant decapitation experiments and the application of exogenous inhibitors of polar auxin transport (PAT) (Beveridge et al., 2000; Fichtner et al., 2017). Two hypotheses have been put forward to explain the release of buds from apical dominance. The first one is the ‘auxin canalization’ hypothesis that states that buds can only grow upon export of auxin. The efflux carrier PIN1 mediates PAT and integrates developmental information along the plant axis (Geldner et al., 2001). Thus, removal of the plant apex would reduce competition between apical and axillary buds to export auxin through PIN1 carriers (Balla et al., 2011). However, recent work has shown that axillary bud growth is independent of auxin upon decapitation of pea plants (Chabikwa et al., 2019). Alternatively, the ‘second messenger’ hypothesis states that another signal must enter the bud to activate it. Cytokinins (CKs) are a potential second messenger influencing bud outgrowth.

CKs act antagonistically to auxin, suppressing apical dominance and releasing buds from dormancy (Ferguson and Beveridge, 2009). Emerging evidence suggests that CKs may influence PAT, as exogenous CK treatment results in increased accumulation of PIN transporters in shoots (Marhavý et al., 2011; Waldie and Leyser, 2018). CKs are synthesized in the roots by two alternative pathways, either isopentenyladenosine-5′-monophosphate (iPMP)-dependent or iPMP-independent (Sakakibara, 2021). CKs, or their precursors, synthesized in the roots and transported acropetally through the xylem could thus reach arrested side buds and break their dormancy. Additionally, auxin modulates CK concentration by repressing its biosynthesis (Ferguson and Beveridge, 2009). CKs are also main controllers of sink establishment (Eviatar-Ribak et al., 2013), a condition necessary to stimulate the vigorous growth of the side shoot. The whole picture of control of branch outgrowth was rendered more complex by the discovery of altered branching pattern mutants that are neither auxin nor cytokinin mutants, including *max* (*more axillary branching*) in Arabidopsis, *rms* (*ramosus*) in pea and *dad* (*decreased apical dominance*) in petunia. Cloning of the *MAX* genes in Arabidopsis showed that *MAX1*, *3* and *4* are involved in the biosynthesis of an acropetally mobile signal, whereas *MAX2* is present in the shoot and acts in the signal transduction of this signal. The mobile compounds are strigolactones, a group of sesquiterpenes derived from carotenoids (Gomez-Roldan et al., 2008; Umehara et al., 2008). Strigolactones promote bud inhibition by modulating auxin transport, and control the amount of PIN transporters in the shoot (Bennett et al., 2006). There is also an interplay between strigolactones and CKs (Dun et al., 2012). For instance, strigolactone-deficient mutants show very low levels of root-derived cytokinins in the xylem sap (Dun et al., 2012). Nutrient deficiency induces the biosynthesis of strigolactones, inhibiting shoot branching (Kapulnik and Koltai, 2014).

Consistently with the model described above, transgenic plants overexpressing CK biosynthesis genes show excessive branching (Medford et al., 1989; Smart et al., 1991; Smigocki, 1995). The bushy aspect of CK overproducer plants suggests that CK depletion could lead to the opposite phenotype, i.e., reduced side branch initiation or increased apical dominance. Although it is difficult to inhibit cytokinin due to the lack of effective inhibitors of its biosynthesis and transport (Eckardt, 2003), its endogenous content can be manipulated in transgenic plants overexpressing the *CYTOKININ OXIDASE* (*CKX*) gene, which encodes for a CK-deactivating enzyme (Werner et al., 2001, 2003). Here we produced transgenic tomato plants overexpressing the Arabidopsis *CKX2* gene (CKX2-OE). The CKX2-OE plants showed delayed vegetative and reproductive development, shorter roots and increased adventitious root growth. The lower CK levels in CKX2-OE plants were also associated with increased branching, which conflicts with CK-overproducing plants also showing a similar phenotype (Medford et al., 1989; Smart et al., 1991; Smigocki, 1995). Grafting, gene expression analysis and double mutant analysis indicate that the increased branching in CKX2-OE plants is unlikely to be mediated by root-derived signals, including strigolactones, although it is linked to a down regulation of the tomato TCP transcription factor *BRANCHED1* (*SlBRC1b*) (Martín-Trillo et al., 2011) and is dependent of the GRAS gene *LATERAL SUPPRESSOR* (*LS*) (Schumacher et al., 1999). On the other hand, decapitation experiments and analysis of PAT evidenced that altered auxin transport from the apex of CKX2-OE plants is likely to be insufficient to inhibit axillary bud growth. Taken together, our results provide evidences that plants with opposite cytokinin status could present excessive branching altering the status of other branching-associated molecules, such as the hormone auxin.

## Results

### Shoot branching and other developmental parameters in *CKX2*-overexpressing tomato plants

**Fig. 1:**
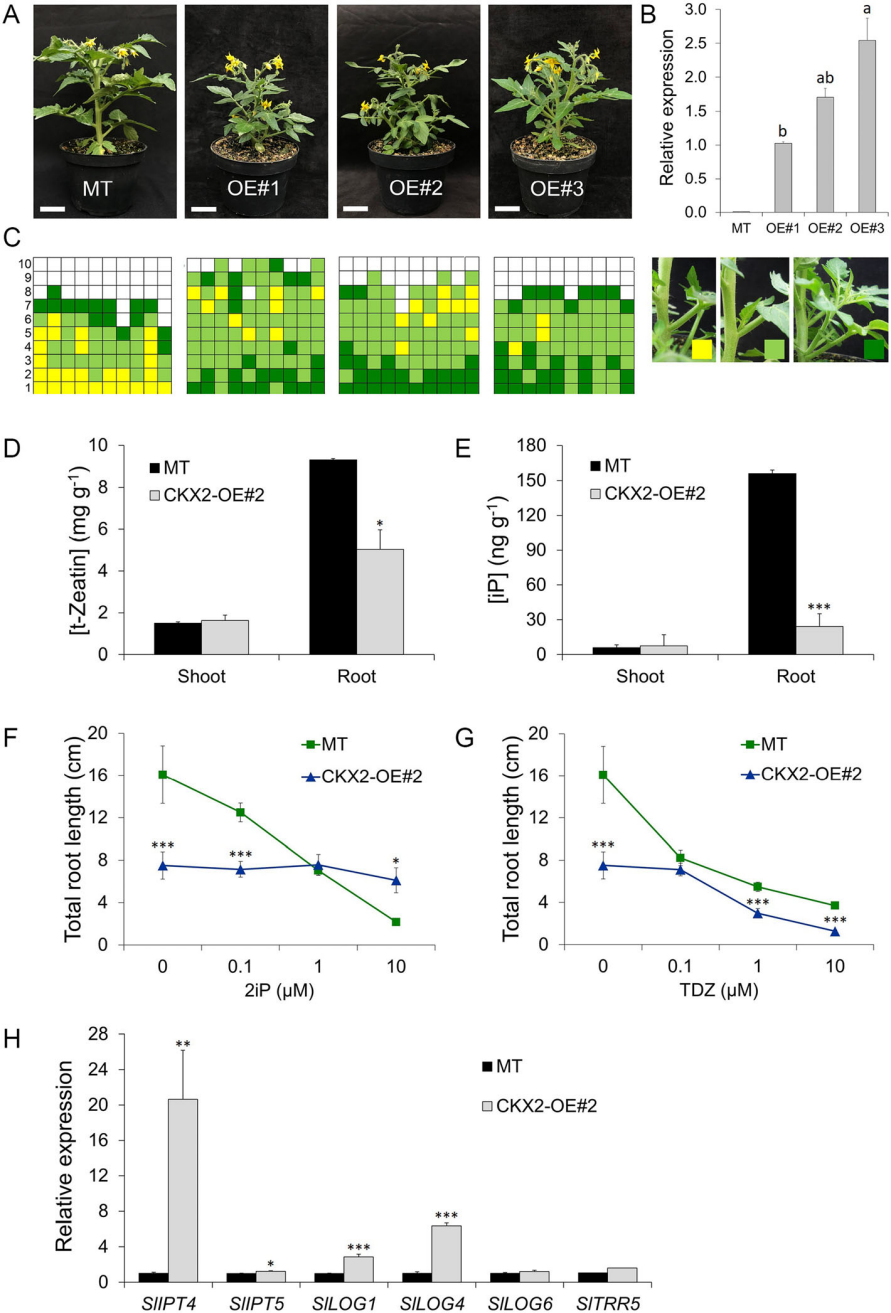
*CKX2*-overexpressing tomato plants display increased shoot branching and alters cytokinin accumulation and responses. **A.** Phenotype of *CKX2* overexpressing (OE) lines (#1, #2 and #3) in tomato Micro-Tom (MT) background. **B.** *AtCKX2* gene expression analysis. Transcript accumulation was estimated compared to CKX2-OE#1 in axillary buds (0.5 to 1.0 cm) harvested from the 3rd and 4th node of vegetative plants. Data are means ± se of three biological samples. Letters indicate significant differences among the transgenic lines. **C**. Schematic representation of axillary shoot branching in the sympodial axis at anthesis. Squares represent individual leaf axils and columns represent single plants. Oldest (#1 at the bottom) to the youngest (#10 at the top) leaves are shown within the column. Gray squares indicate absence of axillary branch; yellow (< 0.5 cm), pale green (≥ 0.5 cm to ≤3 cm) and dark green (> 3 cm) squares indicate growing branches. **D** and **E**. Concentration of endogenous cytokinin t-Zeatin (**A**) and iP (**B**) in vegetative shoots and roots in CKX2-OE#2 and MT plants. Data are means ± se (*n* = 3 for shoot and *n* = 5 for root). **F** and **G**. Root growth inhibition in response to 2iP (**E**) or TDZ (**F**) in CKX2-OE#2 compared with the MT. 2iP, N^6^-(2-Isopentenyl) adenine; TDZ, thidiazuron. Total root length (primary and lateral roots for MT; primary, lateral and adventitious roots for CKX2-OE#2) was measured 7 days after treatment; Data are means ± se (*n* = 12). **H**. Transcript levels of cytokinin-biosynthesis, −activation and -response genes in CKX2-OE#2 and MT shoot buds. Transcript accumulation was measured in axillary buds (0.5 to 1.0 cm) harvested from the 3rd and 4th node of vegetative plants. Asterisks indicate significant differences between genotypes (*P < 0.05**, *P* < 0.01, ***P < 0.001). **B**: Tukey test; **D**, **E** and **H**: Student’s *t*-test; **F** and **G**: Mann Whitney test

Three independent transgenic *CKX2*-overexpression (CKX2-OE) lines (CKX2-OE#1, CKX2-OE#2 and CKX2-OE#3) were generated in the cv Micro-Tom (MT) genetic background. Phenotypic analyses show changes in shoot architecture (Fig. 1A), which did not fully correlate with At*CKX2* expression levels (Fig. 1B), suggesting an indirect effect of CKX overexpression in the three transgenic events. A quantitative analysis of the branching pattern (Fig. 1C) shows that *CKX2-OE* plants have increased shoot branching (*n* = 10). MT plants show an increasing rate of bud outgrowth from bottom (basal) to top (apical) nodes. In the transgenic lines, the shoot branching pattern was altered, whereby most of the upper axillary buds were of intermediate length while most of the basal ones were longer than 3 cm (Fig. 1C). In *CKX2*-OE#3, longer branches were found both in the basal and distal nodes of the shoot (Fig. 1C).

Pleiotropic developmental changes were noticeable during the vegetative developmental phase of all three *CKX2*-OE lines. *CKX2*-OE took longer to reach anthesis and produced more leaves in the primary shoot before the first inflorescence, when compared to MT (Table S1). Despite the larger number of leaves per plant, the total leaf area of the three transgenic lines was reduced in comparison to MT (Table S1). This is due to the small leaflets of *CKX2*-OE plants, which also have altered morphology with smooth margins and lanceolate shape (Fig. S1). Induced expression of CKX3 in transgenic *Arabidopsis thaliana* accelerated leaf senescence (Hu et al., 2021). In contrast, no obvious signs of accelerated leaf senescence were observed in transgenic tomato plants that overexpress CKX2. These results indicate that ectopic expression of *CKX2* in tomato causes retardation of development and affect leaflet morphogenesis.

To dissect the developmental effects described above, we choose the *CKX2*-OE#2 line (OE#2 for brevity), which shows an intermediate *CKX2* transcript accumulation level among the three transgenic tomato lines (Fig. 1B). First, we evaluated seedling emergence from soil (Fig. S2). In MT, the first seedlings emerged four days after sowing (DAS) and the highest emergency frequency (44%) occurs at five DAS. In OE#2, the seedlings started to emerge five DAS, with most of them (50%) emerging at six DAS (Fig. S2), indicating retardation in the initial development of tomato plants. To further examine the impact of *CKX2*-OE in the development of tomato plants, a time course observation of shoot apical meristem (SAM) maturation, and floral transition to inflorescence meristem analyses were coupled with the axillary bud initiation. In determinate tomato cultivars (harboring the *sp* mutation), such as MT, SAM activity terminates at the floral transition by conversion to an inflorescence meristem (Pnueli et al., 1998). We compared the SAM of MT and OE#2, in the vegetative (Fig. S2C and D), transition (Fig. S2E and F) and inflorescence (Fig. S2G and H) stages. We observed that some seedlings of MT transitioned from vegetative to reproductive stage four days after emergence (DAE), and that most of the shoot apex (76%) switched to reproductive (inflorescence) stage at ten DAE (Fig. S2I). For OE#2 seedlings, all shoot apices remained in vegetative stage at 16 DAE, and that the transition stage were found at 28 DAE (Fig. S2J). Interestingly, reproductive (inflorescence) stage was found at 39 DAE and 75% of OE#2 shoots apices reached the reproductive phase only after 46 DAE. Therefore, overexpression of *CKX2* in tomato led to a delay in floral transition. These results agree with the analysis of the number of leaves produced before the first inflorescence (Table S1). At 14 DAE, the first pair of true leaves were already developing in MT seedlings, while at that point, OE#2 showed only cotyledons (Fig. S3). The first pair of true leaves of OE#2 appeared at 28 DAE, by which time MT has four leaves. At 32 DAE, *CKX2*-OE lines have 3–4 leaves compared to five leaves in MT (Fig. S3).

Given that in tomato, the breakdown of apical dominance usually occurs when plants start flowering (Pnueli et al., 1998) and that the apical dominance exerted by the shoot apex affects lateral bud outgrowth (Cline, 1994), we tested whether shoot branching at anthesis is affected by the retarded development by comparing the axillary bud initiation in OE#2 and MT plants (Fig. S2K and L). At 13 and 16 DAE, almost all axillary buds are formed in the axil of MT (Fig. S2M and N). In OE#2 at 13 and 16 DAE, less than a half of leaf axils developed an axillary bud (Fig. S2O and P). At this stage, OE#2 SAM is still vegetative (Fig. S2J), while the MT SAM has already undergone conversion to inflorescence (Fig. S2I), showing that the axillary bud initiation is closely depending on the SAM maturation. In spite of this, the high branching growth of *CKX2*-OE plants could not be attributed to an indirect effect of alterations in the transition from vegetative to reproductive development, which breaks down the apical dominance, since the OE#2 phenotype is the opposite of what is expected as a result of its retarded transition.

In order to investigate the effect of ectopic *CKX2* expression in root development, seedlings of OE#2 and MT were grown in vitro. Primary root length was significantly reduced in OE#2 plants when compared to MT in all stages of development observed (Fig. S4A and B). The total root length of OE#2 was also initially smaller than in MT, but caught up at 16 days after germination (DAG) (Fig. S4C). This increase in total root length in OE#2 is not due to the contribution of the number of lateral roots, which is lower to that of MT (Fig. S4D), but seems to be due to the formation of adventitious roots, which grow profusely in the transgenic lines (Fig. S4E). These results indicate that the root architecture in transgenic plants is distinct to MT, in which the abundance of adventitious roots in *CKX2*-overexpressing tomato plants resulted in a ‘bushy’ root phenotype, indicating that CK deficiency can alter lateral root and adventitious roots development.

To evaluate whether the altered shoot and root growth phenotypes were associated with changes in tissue and cell morphology, a detailed histological analysis was performed comparing OE#2 and MT seedlings. Despite the apparent slower formation of leaf primordia, the general organization and structure of the OE#2 SAM were similar to that of MT, as evidenced in longitudinal sections of the stem (Fig. S5A). Hypocotyls of OE#2 were thinner and with an additional cortical cell layer compared to MT. Moreover, the vessels in the hypocotyl of the transgenic line are unevenly distributed (Fig. S5D). In the primary root, the cross-sections showed smaller cells in OE#2, but without compromising the number of layers in this tissue (Fig. S5F).

### Cytokinin (CK) biosynthesis and responses in CKX2-OE tomato plants

To verify the impact of *CKX2* overexpression on CK status in tomato tissues, we investigated the endogenous CK content, the effect of exogenous CKs and the expression of CK-related genes in OE#2 and MT plants. Either in MT or transgenic plants, the t-Zeatin content was much more abundant than iP derivates in both shoots and roots (Fig. 1D and E), with 1000-fold more t-Zeatin than iP content on a dry weight basis. Both t-Zeatin and iP levels were lower in OE#2 roots compared to MT. In vitro assays showed that CKX2-OE potato plants have reduced content of bioactive forms of cytokinin in both shoots and roots (Raspor et al., 2012). Increased CKX2 activity was also reported in vitro in shoots of centaury (Trifunovic et al., 2013). However, neither t-Zeatin nor iP levels were different between MT and OE#2 in shoots (Fig. 1D and E). These results indicate that ectopic expression of *CKX2* affects mainly CK content in roots, which are the primary source of this hormone (Chen et al., 1985). On the other hand, it is possible that CK sensitivity and signaling is differentially modulated in shoot or root of the transgenic lines.

To measure the CK response in roots, in vitro grown plants were treated with iP and the synthetic CK-analogous thidiazuron (TDZ), which is not a substrate of *CKX2* (Letham and Palni, 1983). Increasing concentrations of iP resulted in root growth inhibition in MT, whereas OE#2 roots showed no alteration in total root length (Fig. 1F). In contrast, increased TDZ concentration resulted in root growth inhibition for both MT and OE#2 plants (Fig. 1G). This result seems to indicate that overexpression of *CKX2* in tomato roots can degrade iP, impairing its effect on root growth. Conversely, the inability to metabolize TDZ by both MT and *OE#2* plants caused root growth inhibition. Moreover, considering that iP, not t-Zeatin, is the preferential substrate of *CKX* enzymes (Mok and Mok, 2001), these results may explain the much lower endogenous content of iP than t-Zeatin, when comparing OE#2 and MT roots (Fig. 1D and E).

We next investigated the expression of CK biosynthesis and response genes in *CKX2*-OE and MT shoot buds. To select CK-related genes with greater expression in the tomato stem, which could directly affect bud outgrowth, we performed an in silico analysis of the gene expression pattern (Zouine et al., 2017). We selected the CK-biosynthesis genes *SlIPT4* and *SlIPT5*, the CK-activating genes *SlLOG1*, *SlLOG4* and *SlLOG6*, and the CK-signaling gene *SlTRR5*. *SlIPT4* transcript accumulation was 20-fold greater in *CKX2*-overexpressing tomato buds compared to MT (Fig. 1H). Transcript levels of *SlIPT5* were slightly higher in OE#2 compared to MT and (Fig. 1H). Expression levels of *SlLOG1* and *SlLOG4* were 2.8 and 6.3-fold greater in OE#2, when compared to MT. On the other hand, the expression of *SlLOG6* and *SlTRR5*, were just slightly higher in OE#2, when compared to MT shoots (Fig. 1H). We also produced MT and OE#2 plants expressing the bacterial *beta-glucuronidase* (GUS) reporter gene under the control of the *ARABIDOPSIS RESPONSE REGULATOR 5* (*ARR5*) promoter, which is a marker of CK status in histochemical analyses (D’Agostino et al., 2000). The GUS stain intensity of OE#2 axillary buds was lower than that of MT (Fig. S6A and B). These findings suggest that a regulatory feedback mechanism may be occurring in the axillary bud region of the *CKX2*-OE plants, resulting in a local higher expression of CK biosynthesis and activation genes to compensate for the lower CK status.

To verify the response of axillary buds to exogenous CK, benzylaminopurine (BAP) was supplied to the leaf axils of vegetative OE#2 and MT plants. BAP application enhanced axillary shoot growth of both MT and OE#2 plants (Fig. S7). However, the effect of BAP application was less significant in OE#2 plants than in MT, suggesting that *CKX2*-OE buds have a lower response to exogenous CKs.

### Shoot branching in reciprocal grafts between *CKX2*-OE and MT plants

**Fig. 2:**
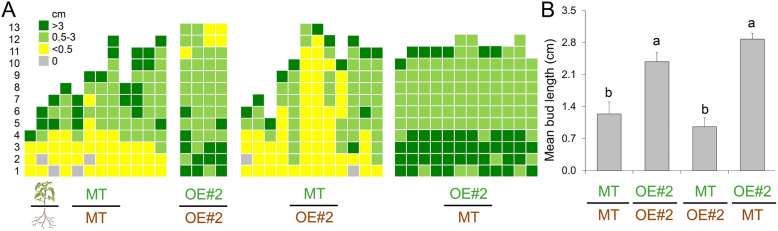
Increased shoot branching in CKX2-overexpressing plants is not caused by a root-derived signal. **A.** Schematic representation of axillary shoot branching of grafted genotypes at anthesis. **B**. Mean bud length of CKX2-OE#2 (here described as OE#2) and MT plants. Data are means ± se. Different letters indicate significant differences (Tukey test, *P* < 0.05) among grafted plants

To test whether the decreased endogenous root-produced CK in *CKX2*-OE tomato plants could affect axillary bud outgrowth in the shoot, we performed reciprocal grafting between *CKX2*-OE and MT plants. In vitro grown seedlings at the same physiological stage, i.e. nine days after sown (DAS) for MT and 13 DAS for OE#2, were used for reciprocal micrografting (Fig. 2). Both grafted controls showed the same branching pattern observed in intact MT and OE#2 plants (Fig. 1A, C), in which MT/MT showed high apical and low basal branching, while in OE#2/OE#2 grafts the most intense outgrowth was observed in basal nodes (Fig. 2A). When an MT scion was grafted onto an OE#2 rootstock, no difference was observed in the shoot branching pattern (Fig. 2A), neither in the mean bud length (Fig. 2B), compared to MT/MT plants. A slight increase in the mean bud length was observed in OE#2 scion grafted onto MT rootstock compared to OE#2/OE#2, though not statistically significant (Fig. 2B). These results indicate that the increased branching in *CKX2*-OE is unlikely to be mediated by a root-derived component.

### The effect of *LATERAL SUPPRESSOR* (*LS*) and *BRANCHED1* (*BRC1*) genes on shoot branching in *CKX2*-OE plants

**Fig. 3:**
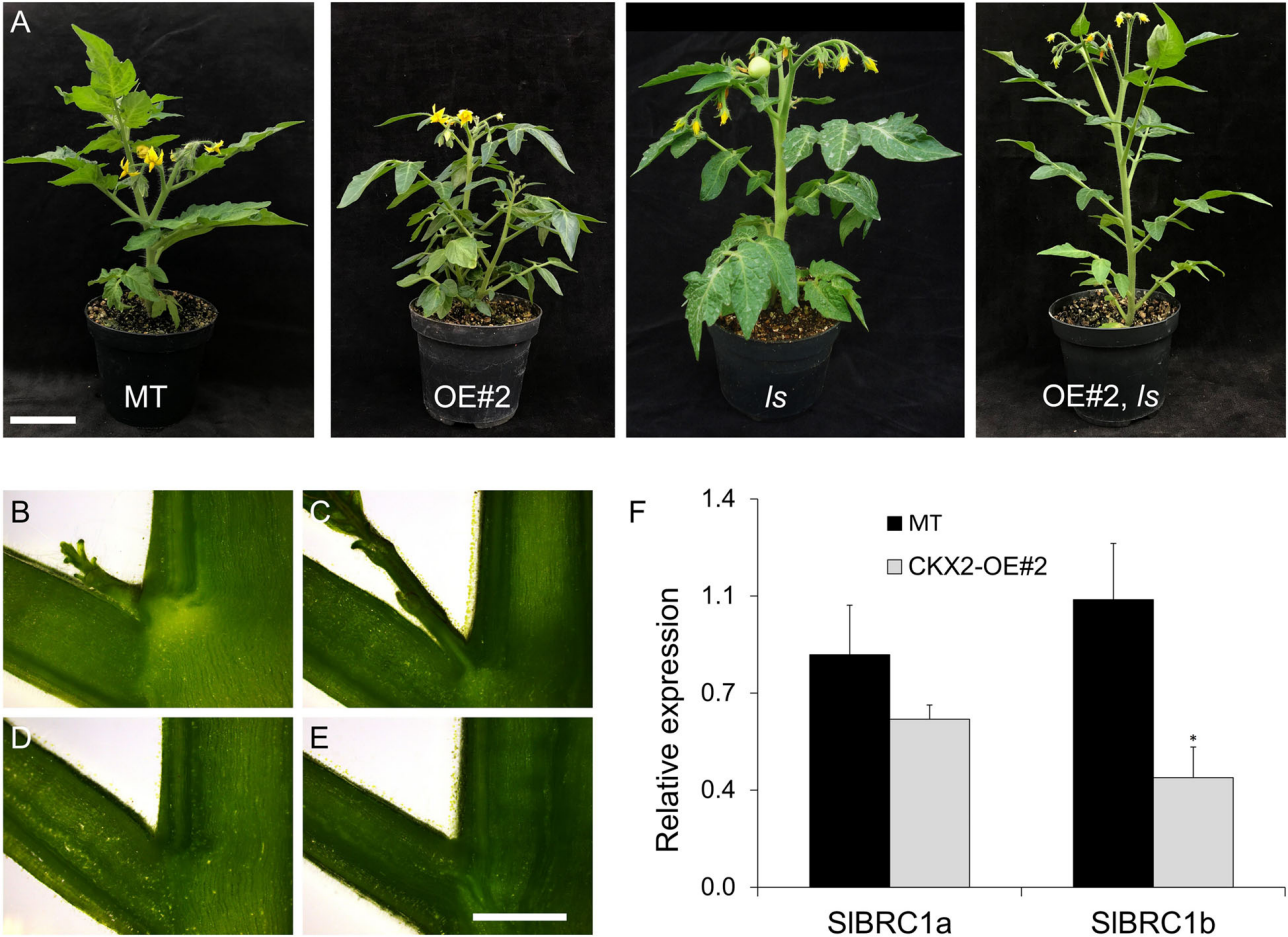
Shoot branching in CKX2-overexpressing plants is dependent on functional *LS* and linked to *BRC1* repression. **A.** Representative phenotype of plants of MT, CKX2-OE#2, *ls* mutant and the double transgenic-mutant CKX2-OE, *ls*. **B to E**. Longitudinal shoot section of MT (**B**), CKX2-OE#2 (C), *ls* (**D**) and CKX2-OE, *ls* (**E**) leaf axil; bar = 2 mm. **F**. Expression levels of *SlBRC1a* and *SlBRC1b* in axillary buds (0.5 to 1.0 cm) harvested from the 3rd and 4th nodes of vegetative plants of MT and CKX2-OE#2. Data are means ± se of three biological samples. Statistical comparisons shown were made between the MT and transgenic line (Student’s *t*-test; *P* < 0.05)

We next crossed the *CKX2*-OE tomato plants with the *lateral suppressor* (*ls*) mutant to investigate if the mechanism behind the high bud initiation and outgrowth in *CKX2*-overexpressing tomato plants was dependent on the *LS* gene, which controls axillary meristem formation in tomato (Schumacher et al., 1999). Homozygous progeny of the double transgenic-mutant OE#2, *ls* showed a similar leaflet phenotype as OE#2 plants, *i.e*, lanceolate with smooth margins. However, branching was strongly suppressed, resembling the *ls* mutant (Fig. 3). Although neither *ls* nor OE#2 developed lateral buds, they formed the characteristic lateral branch in the axil of the last leaf under the inflorescence of the primary shoot, which corresponds to the sympodial branch in MT. Longitudinal section of the stem in the leaf axil region showed the growing bud in MT (Fig. 3B), OE#2 (Fig. 3C) and its absence in the *ls* (Fig. 3D) and OE#2, *ls* (Fig. 3E). We also determined the expression pattern of *S. lycopersicum BRANCHED1a* (*SlBRC1a*) and *SlBRC1b,* which are related to axillary meristem formation and bud outgrowth in tomato (Martín-Trillo et al., 2011). Transcript accumulation of both genes was lower in OE#2 compared to MT (Fig. 3F). *SlBRC1b*, which has a major role in the control of shoot branching in cultivated tomato (Martín-Trillo et al., 2011), was expressively down-regulated in *CKX2*-OE plants, compared to MT. Altogether, these results suggest that the *CKX2*-OE branching phenotype is dependent of a functional *LS* gene and that it involves a reduced transcript accumulation of *SlBRC1* genes.

### Effect of the inhibition of strigolactone biosynthesis on the shoot branching phenotype of *CKX2*-OE plants

**Fig. 4:**
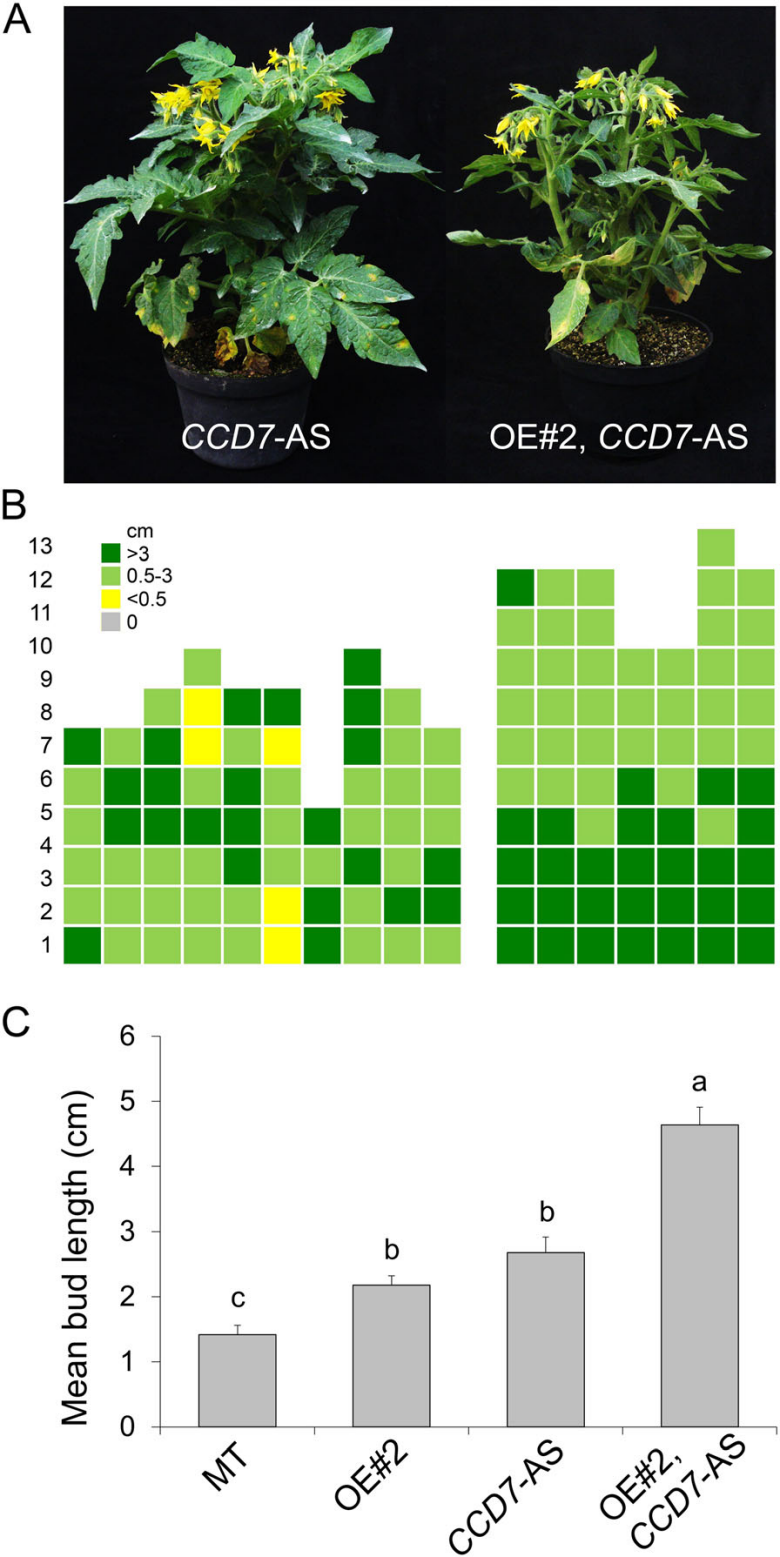
Strigolactone depletion increases shoot branching in tomato plants overexpressing CKX2. **A**. Representative phenotype of antisense for *CCD7* gene (CCD7-AS) and the double transgenic OE#2, CCD7-AS (OE#2 was used here as an abbreviation of CKX2-OE#2). **B.** Schematic representation of axillary bud formation in leaf axils in the sympodial axis of CCD7-AS and CKX2-OE#2, CCD7-AS plants at anthesis. **C**. Mean bud length of MT, CKX2-OE#2, CCD7-AS and CKX2-OE#2, CCD7-AS plants. Data are means ± se. Letters indicate significant differences between genotypes (Tukey test; *P* < 0.05)

The high shoot branching in OE#2 can be caused by changes in the homeostasis of other hormones, such as strigolactones (SLs), root-derived inhibitors of axillary bud growth (Gomez-Roldan et al., 2008; Umehara et al., 2008). We produced a double transgenic genotype crossing OE#2 plant with a MT line expressing an antisense RNAi construct for the *SlCCD7* gene (*CCD7*-AS), which encodes a carotenoid cleavage enzyme necessary for SL biosynthesis (Vogel et al., 2010). Both the single transgenic *CCD7*-AS and the double transgenic OE#2, *CCD7*-AS lines showed a highly branched phenotype (Fig. 4A). The double transgenic line differed from the *CCD7*-AS line in having larger axillary buds in basal nodes (leaf axils #1 to 5) (Fig. 4B). The mean bud length in the double transgenic line was significantly increased when compared to either of the single transgenic lines, indicating an additive interaction (Fig. 4C). These results suggest that the branching phenotype of *CKX2*-OE plants belongs to an additional mechanism complementary to that mediated by SLs, which agrees with the results showing that *CKX2*-OE branching is unlikely to be mediated by a root-derived component (Fig. 2).

### The effect of disturbed auxin status on the shoot branching of *CKX2*-OE tomato plants

**Fig. 5:**
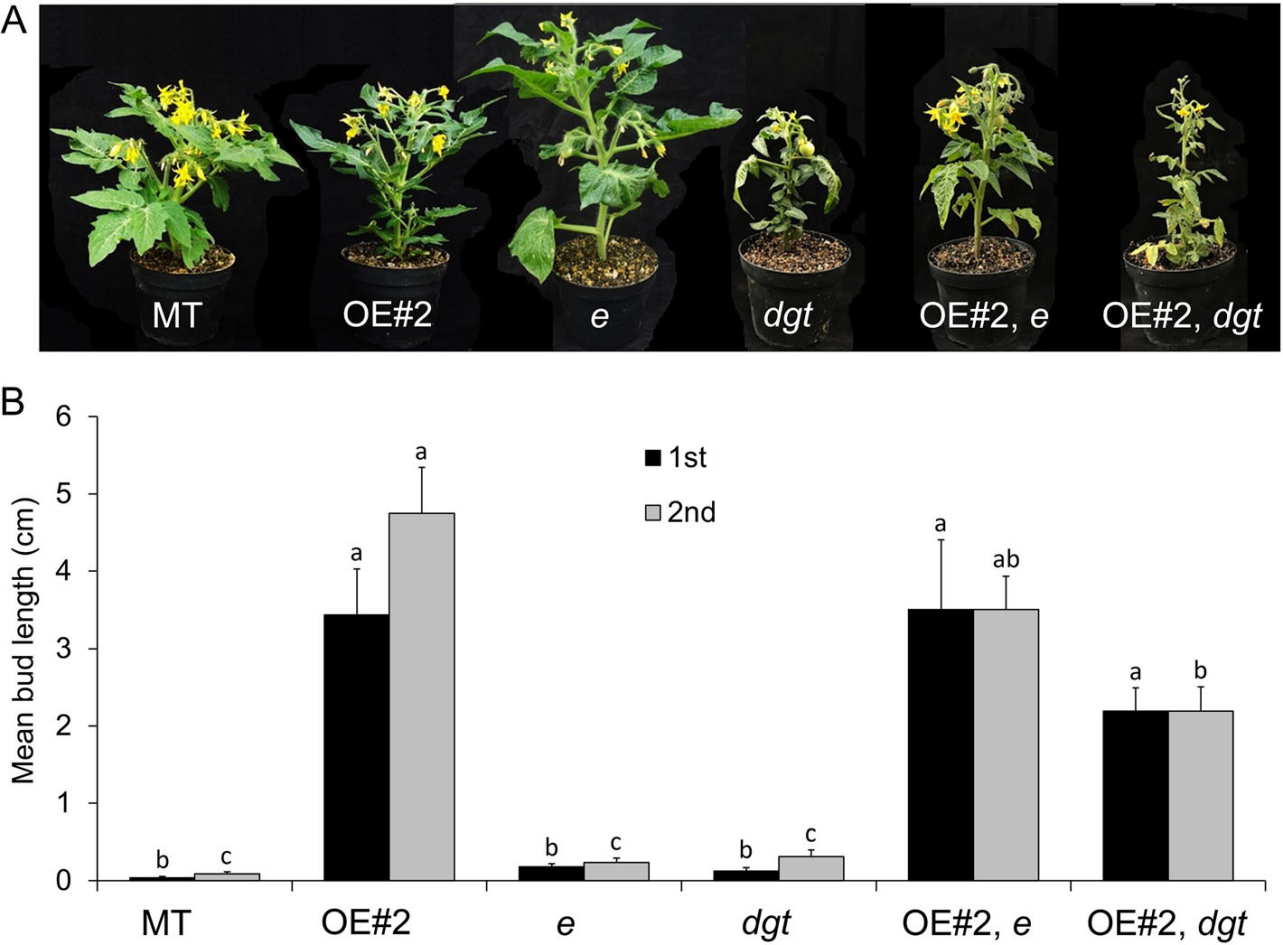
Altered auxin status affects shoot branching in *CKX2*-overexpressing tomato plants. **A.** Shoot phenotype of MT, CKX2-OE#2 (abbreviated as OE#2), *entire* (*e*), *diageotropica* (*dgt*) and the double transgenic-mutant CKX2-OE#2, *e* and CKX2-OE#2, *dgt* plants. **B**. Mean bud length of the first (1st) and second (2nd) axillary bud formed in the bottom at anthesis. Data are means ± se. Letters indicate significant differences between genotypes (Tukey test; *P* < 0.05)

To determine whether the high shoot branching in *CKX2*-OE tomato plants can be caused by alterations on auxin, a major hormone controlling shoot branching (Domagalska and Leyser, 2011), the *entire* (*e*) and *diageotropica* (*dgt*) auxin mutants were crossed with OE#2 generating two double transgenic-mutant lines: OE#2, *e* and OE#2, *dgt*. The *e* mutant has a constitutive response phenotype to auxin, since it is a loss-of-function of the tomato *AUX/IAA9* (*SlIAA9*) gene, which acts as a negative regulator of auxin signaling (Zhang et al., 2007). The *dgt* mutant is a loss-of-function of the tomato *CYCLOPHILIN1* gene (*SlCYP1*; Oh et al., 2006) required for the effective transport of auxin and regulation of *PIN* transporters (Ivanchenko et al., 2015). Homozygous double transgenic-mutant OE#2, *e* and OE#2, *dgt* plants showed strong reduction of shoot development (Fig. 5A), when compared to OE#2 plants. A reduction in bud length (Fig. 5B) was observed in OE#2, *e* and OE#2, *dgt* plants when compared to OE#2. These results indicate that the altered shoot branching observed in OE#2 plants may be modulated by an auxin-dependent mechanism, including the alteration of auxin sensitivity and transport. Reinforcing this hypothesis is the reduced GUS staining in shoot buds of *DR5::GUS*, *CKX2-OE* double transgenic plants when compared to *DR5::GUS* plants (Fig. S6C and D), which are used to track the auxin status in different tissues (Ulmasov et al., 1997).

**Fig. 6:**
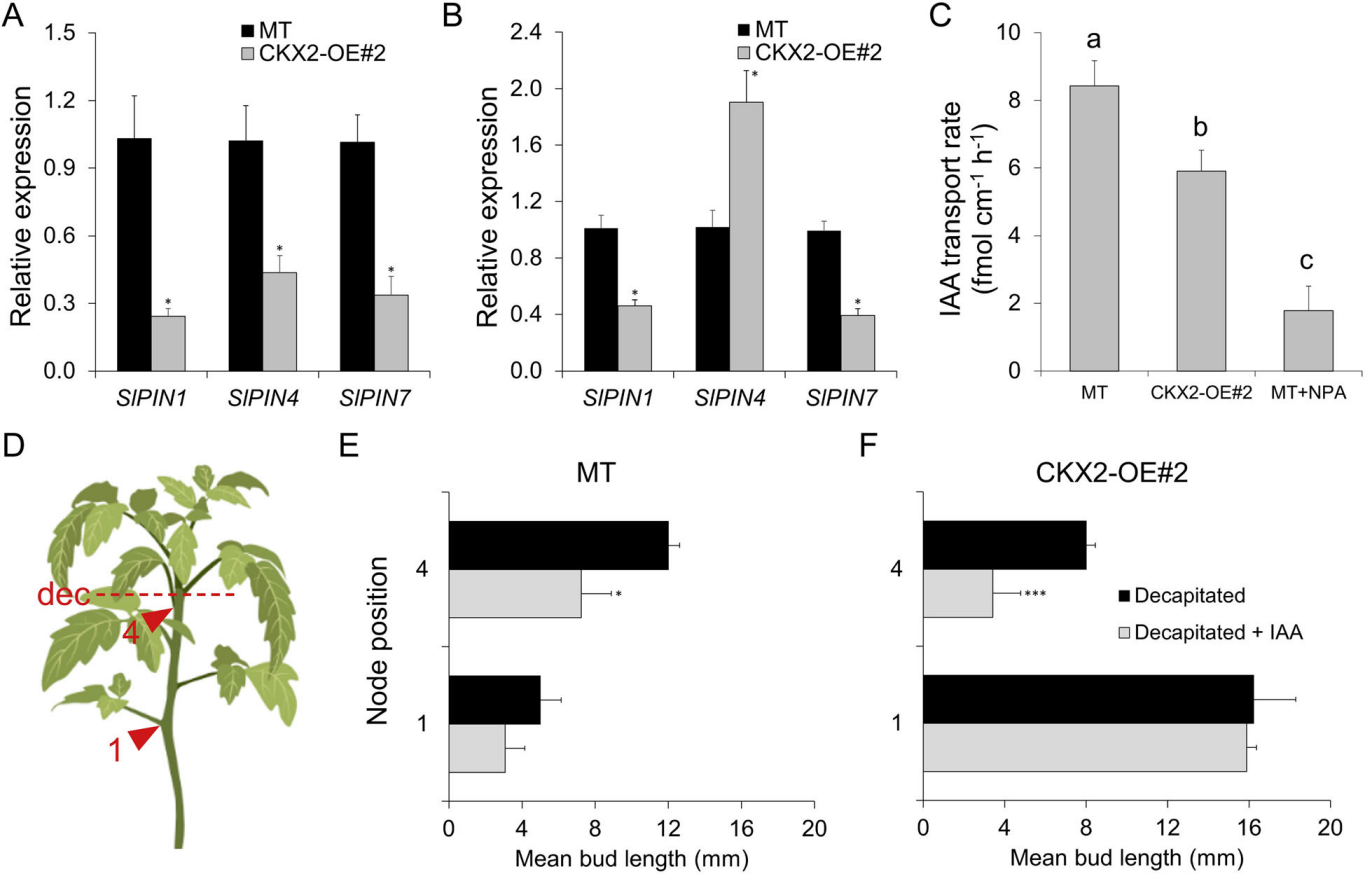
Cytokinin-mediated shoot branching in *CKX2*-overexpressing tomato plants is mediated by a reduced auxin transport. **A** and **B**. Expression levels of *PIN* auxin efflux facilitator genes (*SlPIN1, SlPIN4* and *SlPIN7*) in axillary buds (0.5 to 1.0 cm) harvested from the 3rd and 4th node of vegetative MT and CKX2-OE#2 plants (**A**) and in hypocotyl from 2-week-old seedlings (**B**). Data are means ± se of three biological replicates. **C**. Reduced basipetal auxin transport in CKX2-OE#2 compared to MT plants. Auxin ([^3^H]IAA) transport was measured in 10-mm hypocotyl sections of MT, CKX2-OE#2 and the experimental control (MT treated with 1-Naphthylphthalamic acid [NPA]). Data are means ± se (*n* = 10). **D**. Schematic representation of node position used in the decapitation experiment. **E**. Mean axillary bud length 10 days after decapitation in MT plants. **F**. Mean axillary bud length 10 days after decapitation in CKX2-OE#2 plants. Auxin, IAA (10 μM) was applied to the cut stem stump immediately after decapitation. Data are means ± se (*n* = 12). Letters indicate significant differences between treatments within the same genotype and node position. A and B: Student’s *t*-test; C: Tukey test, *P* < 0.05; E and F: Mann Whitney test; (*P < 0.05, ***P < 0.001)

To assess the role of auxin transport in OE#2 shoot branching, we explored the transcriptional modulation of *PIN-FORMED* (*PIN*) genes, and we measured polar auxin transport (PAT) rate in the stem and auxin response in decapitated plants. PINs belong to transmembrane proteins family of auxin transport facilitators mediating PAT. We analyzed the transcript accumulation of *SlPIN1*, *SlPIN4* and *SlPIN7* in axillary buds (0.5 to 1.0 cm) collected from the third and fourth node of vegetative plants and in hypocotyls from 2-week-old seedlings. Transcript accumulation of all *PIN*s analyzed in axillary buds was significantly reduced in OE#2 compared to MT (Fig. 6A), indicating a lower auxin transport in buds of OE#2 plants. Except for the increase in *SlPIN4* transcripts in OE#2 compared to MT, transcript accumulation of *SlPIN1* and *SlPIN7* was also reduced in OE#2 hypocotyls compared to MT (Fig. 6B). We next determined the auxin transport rate in MT and OE#2 in hypocotyl segments. Compared with MT, the transport rate in OE#2 was significantly lower (Fig. 6C), which agrees with the *PIN* expression results.

To better correlate the reduced *PIN* expression and auxin transport with the increased shoot branching phenotype in OE#2 plants, the impact of exogenous auxin treatment in the shoot branching of decapitated plants was evaluated. Since auxin is mainly produced in the shoot apex (Ljung et al., 2001) and transported from the tip to the base (basipetally) through *PIN1* carriers (Wiśniewska et al., 2006), we measured the bud length of the farthest (first) and the nearest (fourth) node from the shoot apex (Fig. 6D) upon exogenous auxin application in the decapitated shoot apex. The rationale for this experiment is that the node position along the stem could interfere in the availability of auxin to repress the axillary bud outgrowth, reflecting differences in the basipetal auxin transport capacity. Distinct effects of decapitation and auxin application on bud outgrowth in MT and OE#2 plants were observed (Fig. 6E and F). In MT, longer buds developed in the fourth node, compared to the first node (Fig. 6E), regardless the auxin application. In auxin treated MT plants, a repression on bud outgrowth was observed for both the first and the fourth node, when compared to the equivalent nodes of control decapitated MT plants without auxin application (Fig. 6E). This demonstrated that the auxin treatment was effective to suppress bud outgrowth. Conversely, the first node of both auxin-treated and non-treated decapitated OE#2 plants showed longer bud length compared with the fourth node of this genotype (Fig. 6F). Moreover, auxin application in the apex affected the bud outgrowth of the fourth but not of the first node of decapitated OE#2 plants. These results corroborate the existence of impaired auxin transport in *CKX2*-OE plants, which consistently had less effect on the inhibition of bud outgrowth of the farthest bud node.

## Discussion

Side branching is a critical trait for horticultural crops, as it affects plant growth and demands labour-intensive pruning operations. Side branches are generally undesirable due to their effect on plant self-shading, competition for nutrient allocation and reduction in yield. In order to explore the consequences of reduced CK content shaping tomato plant architecture, here we have engineered transgenic tomato (*Solanum lycopersicum*) cv. Micro-Tom (MT) plants with constitutive expression of the gene encoding the CK-inactivating enzyme *CKX2* (*CKX2*). The main phenotypic effects of manipulating this gene were originally described in the model species Arabidopsis (Werner et al., 2003). However, although both tomato and Arabisopsis CKX-OE plants coincide in having increased seed weight (Fig. S8, Werner et al., 2003), their pattern of shoot and root development showed considerable differences. Due to Arabisopsis monopodial, rosette growth habit, it is difficult to extrapolate the alterations in the Arabidopsis shoots to other eudicots. In contrast to Arabidopsis, tomato has a sympodial growth pattern, whereby the conversion of the shoot apical meristem (SAM) into an inflorescence meristem is followed by the vigorous development of the subtending axillary meristem to continue vertical growth (Périlleux et al., 2019). This motif is repeated indefinitely in indeterminate varieties, whereas the *self-pruning* mutation causes premature termination after two consecutive inflorescences are formed (Pnueli et al., 1998). The *sp* mutation divides the tomato crop in two general agronomical types: indeterminate varieties, bred for fresh tomato production, generally in highly controlled greenhouse conditions; and determinate varieties for industrial processing (production of ketchup, sauces) grown in the open field (Robbins et al., 2011). Both processing and fresh market tomato varieties can benefit from reduced side branching since it allows increased planting density in processing tomato and reduces intensive management input in the form of staking and pruning in fresh tomatoes (Zahara, 1970; Navarrete and Jeannequin, 2000).

The tomato Micro-Tom (MT) cultivar carries the recessive allele *sp*, which leads to a determinate growth habit (Marti et al., 2006; Carvalho et al., 2011). Analysis of shoot branching in MT and in the *CKX2*-OE lines at anthesis revealed an increased shoot branching in transgenic tomato plants, which is the opposite effect of what was expected from lower CK content, as CKs are known to stimulate bud outgrowth (Ferguson and Beveridge, 2009). Further, the pattern of bud release and outgrowth along the stem were also altered in the *CKX2*-OE tomato plants. More axillary branches were also observed in *35S:AtCKX1* and *35S:AtCKX3* Arabidopsis transgenic plants after anthesis but no analysis of shoot branching was reported (Werner et al., 2003). Conversely, as expected for CK defective mutants, *ipt3,5,7* triple mutant exhibits decreased shoot branching possible due to defects in bud initiation, since its buds can grow normally (Müller et al., 2015). This raises the question whether a higher CK degradation in *CKX*-overexpressing tomato plants triggers a compensatory mechanism by feedback regulation of the pool of active CKs in shoots or roots. Moreover, part of this compensatory mechanism could also involve alterations in other plant hormones controlling shoot branching.

The three main hormone classes that influence side branching are auxins, cytokinins (CKs) and strigolactones (SLs) (Ferguson and Beveridge, 2009). Auxin depletion in the stem and export from buds are required to sustain bud outgrowth, but not initial bud formation or release from dormancy (Wang et al., 2014b). Root-derived CKs and SLs may induce or suppress bud outgrowth, respectively (Dun et al., 2012). Hormones may exert their effects by a combination of metabolism (synthesis and degradation), transport and signaling. Our results, suggest that CKs cause their paradoxical effects on branching *per alia*, such as altering the status of strigolactones or auxin. Grafting experiments and double transgenic analyses with SL-deficient plants do not support the hypothesis that the phenotype of CKX2-OE plants is due to alterations in SL. On the other hand, we provide evidence supporting a causative link between auxin transport defects and shoot branching in the CK-deficient tomato plants. Firstly, significant reductions in bud length were observed in the double transgenic-mutant *CKX2*-OE, *dgt* and *CKX2*-OE, *e*. These results show that altered basipetal auxin transport in the main stem of *dgt* (Ivanchenko et al., 2015; Silva et al., 2018) or auxin sensitivity in *e* (Wang et al., 2005; Zhang et al., 2007) interfere in shoot branching of CK-deficient tomato plants. Secondly, reduced transcriptional levels of the auxin export-related genes *PIN1*, *PIN4* and *PIN7* in buds and *PIN1* and *PIN7* in hypocotyls were associated with lower auxin transport in stem of intact tomato CKX2-OE plants. In Arabidopsis, PIN1 is the major driver of polar auxin transport in the stem, while PIN3, PIN4, and PIN7 act to disperse auxin from the PATs into the other tissues, including axillary buds (Bennett et al., 2016). Thirdly, inhibition of bud outgrowth in CKX2-OE is less responsive to the application of auxin in decapitated plants, with a lower inhibition of bud outgrowth on the farthest bud node, which well correlates with reduced auxin transport in the shoot.

Considering that an enhanced CK pulse in leaf axil is required during the axillary meristem initiation (Wang et al., 2014a), and therefore, the branching phenotype in *CKX2*-OE plant could promote functional axillary meristem establishment, we assess if the mechanism behind the elevated shoot branching phenotype in *CKX2*-overexpressing tomato plants was dependent on the GRAS-domain transcript factor *LATERAL SUPPRESSOR* (*LS*), a major regulator of axillary meristem initiation required to maintain the meristematic potential of cells (Greb et al., 2003; Schmitz and Theres, 2005). The absence of bud in the leaf axils of the double transgenic-mutant *CKX2*-OE, *ls* as well as in the *ls* mutant confirm the requirement of a functional *LS* for axillary meristem initiation, as previously reported in tomato (Schumacher et al., 1999), Arabidopsis (Greb et al., 2003) and rice (Li et al., 2003). Further, our analysis of axillary bud formation in leaf axils at early stages of vegetative development revealed that, relative to MT plants, *CKX2*-OE has reduced axillary meristem initiation, whereas bud initiated at anthesis is comparable to MT, which is consistent with the fact that axillary meristem and axillary bud formation in CKX2-OE is not compromised but delayed. Thus, the higher branching in *CKX2*-OE tomato plants is probably not due to axillary meristem initiation but rather in bud activation and outgrowth.

A highly branched phenotype was described in *brc1* mutants of Arabidopsis (Aguilar-Martinez et al. 2007) and pea (Braun et al., 2012), indicating that *BRC* genes prevent bud outgrowth. *BRC1* transcripts repression can be locally regulated in buds by CKs (Braun et al., 2012; Dun et al., 2012; Seale et al., 2017) and it also acts downstream to strigolactones (Braun et al., 2012). In transgenic tomato *CKX2*-OE, the two BRC1-like paralogues, *SlBRC1a* and *SlBRC1b*, were locally down-regulated upon bud activation in comparison to MT. In addition, the increased repression of *SlBRC1b* compared to *SlBRC1a* in *CKX2*-OE agreed with the major function of *SlBRC1b* in the control of shoot branching in tomato (Martín-Trillo et al., 2011). These observations are consistent with a role of CK-dependent signaling acting locally in the regulation of *SlBRC1a* and *SlBRC1b* in the control of axillary bud growth in *CKX2*-OE and this would adjust the local activation threshold, allowing branches to activate.

In addition to shoot phenotype, an interesting effect of *CKX2* overexpression was found in the root system, namely the reduction of lateral root formation and the profuse development of adventitious roots. This contrasts with the increased lateral root formation in CKX-OE Arabidopsis plants (Werner et al., 2003). CK and auxin are the major hormones controlling root development and have antagonistic action in this process (Bellini et al., 2014; Bhalerao et al., 2002). CK inhibits lateral root initiation and development (Li et al. 2006; Fukaki and Tasaka 2009). Density of lateral roots is reduced in CK overproducing mutants or by CK application, whereas lateral root organogenesis is enhanced by decreased CK activity (Li et al. 2006). Initiation and subsequent lateral root development require auxin transport (Bhalerao et al., 2002). Decreased free IAA levels in roots, after applying *N*-1-naphthylphthalamic acid (NPA), a chemical inhibitor of PAT, reduced the number of lateral roots (Casimiro et al., 2001). In the same way, mutants with altered PAT showed disturbed lateral root initiation (Benková et al. 2003). Given the opposite role of auxin and CK in root development and the root phenotype of *CKX2*-OE tomato plants, we hypothesize that the reduction in lateral root formation in the transgenic line is an indirect effect of CK in the polar auxin transport, which is reduced in *CKX2*-OE compared to MT. On the other hand, the abundance of adventitious roots in *CKX2*-OE tomato plants resulted in a bushy root phenotype. The formation of adventitious roots in shoots depends on more complex events, such the acquisition of competence to assume novel developmental fates (Lombardi-Crestana et al., 2012). Therefore, the profuse formation of adventitious roots in CKX2-OE plants observed here deserves further investigation of the molecular mechanism involved, such as the expression of genes related to cell identity. Independent of the molecular mechanism behind the formation of adventitious roots in CKX2-OE plants, its biotechnological potential could be also explored (Ghanem et al., 2011).

**Fig. 7:**
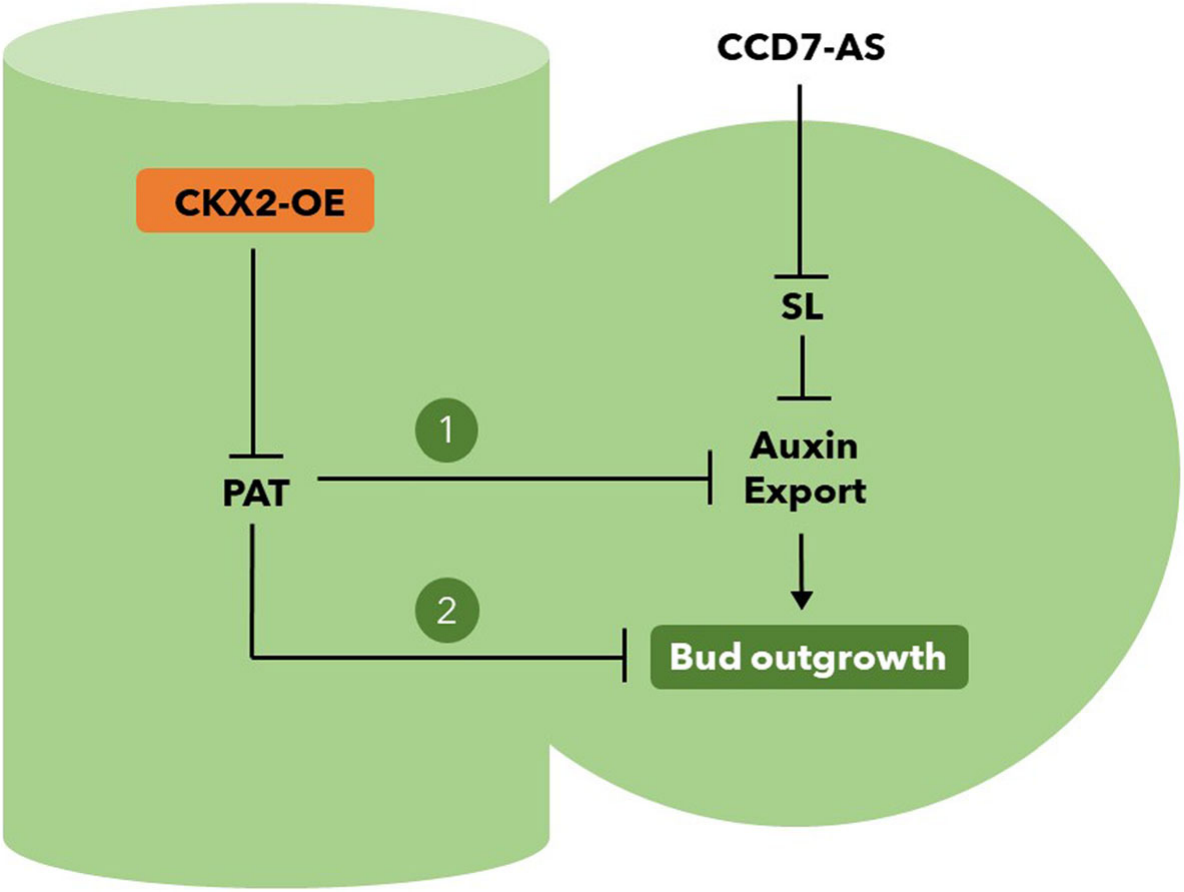
Working model for the role of CKX2 in tomato shoot branching. The inhibition of polar auxin transport (PAT) in the stem by the overexpression of *CKX2* (CKX2-OE) could release bud outgrowth mediated (1) or not (2) by auxin export in the bud. It is well known that PAT competitively inhibits auxin export from the axillary buds, which is a prerequisite for bud outgrowth (Barbier et al., 2018). Strigolactone (SL) also inhibits auxin export, which can be reverted in CCD7-AS plants. The additive phenotype of the double transgenic CKX2-OE, CCD7-AS plants (see Fig. 4) could be the consequence of the magnification of the convergent realease of auxin export (1) or due to another unknown mechanism (2) where CKX2-OE and CCD7-AS plants act in parallel pathways controlling bud outgrowth

In conclusion, our study demonstrates that CKs play an important role in regulating tomato plant architecture, but that their effects on lateral shoot branching are strongly pleiotropic and include changes in meristem maturation and root growth. We have furthermore shed new light on the complexity between auxin, CKs and SLs in the control of axillary branching (summarized in Fig. 7). This working model could additionaly involve a series of epistatic effects between signaling genes and transcription factors. Thus, our results demonstrate that a straightforward pathway to biotechnological manipulation of side branching in tomato is not yet possible without further exploration of the molecular machinery controlling bud formation and outgrowth. Understanding the intricacies of the hormonal control of axillary meristems remains a key step to achieve the highly desirable agronomic aim of controlling side branching in horticultural crops.

## Methods

### Plant material and growth conditions

The tomato cv Micro-Tom, near isogenic lines (NILs) *dgt*, *e* and *ls* mutants, antisense transgenic line for CCD7, CKX2-overexpressing plants (CKX2-OE) and *ARR5::GUS* and *DR5::GUS* lines, all in the same genetic background (cv. Micro-Tom, MT), were used in the experiments. *Dgt* and *e* were obtained as described previously (Carvalho et al., 2011) and *ls* was introgressed into MT as described for the Rg1 allele (Pino et al., 2010). The *ARR5::GUS* line was obtained by genetic transformation of MT using a construct (D’Agostino et al., 2000) obtained from Dr. JJ Kieber (University of North Carolina, USA). The *DR5::GUS* line in MT was obtained from Dr. Jose Luis Garcia-Martinez (Universidad Politécnica de Valencia, Spain). *AtCKX2* under the transcriptional control of the CaMV35S promoter cloned into the pROK2 vector (Alonso et al., 2003) was used to generate the CKX2-OE lines in MT background. M82 seeds carrying *pDESTOE*::*CCD7* antisense construct (Figwort mosaic virus promoter, Richins et al., 1987) were obtained from Dr. Harry Klee (University of Florida). The M82 transgenic plants expressing the *SlCCD7* antisense construct (CCD7-AS) display greatly increased branching (Vogel et al., 2010). This construct was introgressed into cv. Micro-Tom, by successive backcrosses into MT until BC6Fn, as described previously (Pino et al., 2010). After every two backcrosses (BC), plants were self-pollinated resulting in BC2F2, BC4F2 and BC6F2. In F2 and each BCnF2, recombinants showing kanamycin resistance and MT small size were selected. At the anthesis, the increased shoot branching was used as a morphological marker for the transgene in kanamycin resistant plants. In addition to these two selection methods, in BC6F2 and BC6F3 selected transgenic plants were confirmed by PCR. MT plants harboring the CCD7-AS transgene were crossed with CKX2-OE#2. F1 progeny was self-pollinated. F2 plants were screened the presence of both CCD7-AS and CKX2-OE insertion by PCR. For CCD7-AS, we used a primer that amplify a fragment of the FMV promoter (5′- AGTCCAAAGCCTCAACAAGGT-3′; and 3′- TCGTCACTGCGTTCGTCATA-5′), whose sequence was kindly provided by Prof. Harry Klee. For CKX2-OE screening, a primer that amplify a fragment of *AtCKX2* (5′-GGACCATGCACCTAAACGGG-3′; and 3′-TCTCCCCATCATCAGCAAGGT-5′) was used. Plants were grown under standard greenhouse conditions, as previously described (Vicente et al., 2015).

### Plant transformation and culture conditions

Genetic transformation of tomato cv. MT was performed as previously described (Pino et al., 2010). We used cotyledons from 8-days-old seedlings as explants and the *Agrobacterium* strain GV3101. Agrobacterium suspension was dripped on the explants using a sterile pipette. After 10 min the bacterial suspension was removed and explants were blotted dry on sterile filter paper. Plates were dark-incubated at 28 °C for 2 days for co-cultivation. After that, explants were transferred to MS supplemented with B5 vitamins, 30 g/l sucrose, 5 μM 6-Benzylaminopurine (BAP), 100 mg/l kanamycin, 300 mg/l Timentin and 7 g/l agar. Explants were cultivated in growth room under 16 h photoperiod at 25 ± 1 °C for 3 weeks. To allow root elongation, well-developed shoots were detached from the explants and transferred to flasks containing fresh hormone-free MS, supplemented with 100 mg/l kanamycin and 300 mg/l Timentin. Rooted plantlets were acclimated in the greenhouse. Transgenic T_0_ plants were allowed to self-pollinate. Four independent kanamycin-resistant transgenic lines were selected, and three were further evaluated at T_3_ generation or subsequent generation. Genomic DNA was extracted from young leaves as previously described (Fulton et al., 1995) in the T_2_ and T_3_ progeny to confirm the T-DNA integration by PCR.

### Isolation of genomic DNA

Genomic DNA was extracted according to the protocol originally described by Fulton et al. (1995), with some modifications. One or two young leaflets (50 to 100 mg) were collected from vegetative plants and stored in a microtube (1.5 ml) at liquid nitrogen. Using a plastic pestle, frozen leaves were manually macerated in the microtube. Microprep buffer (750 μl) was added to each tube, and the tissue was crushed for more 1 min. Samples were vortexed and incubated at 65 °C for 60 min in water bath. In the same tube, 600 μl of chloroform/isoamyl alcohol (24:1 v/v) was added to each sample. Samples were vortexed briefly and after that centrifuged for 5 min at 10,000 rpm using a microcentrifuge. Aqueous phase (supernatant) was transferred to a new 1.5 ml tube and 600 μl of ice-cold isopropanol was added to the tube, which was inverted 5 times to precipitate the DNA. Samples were centrifuged at 10,000 rpm for 5 min and the supernatant was discarded. The DNA pellet was observed deep in the tube. Ethanol 70% (1 ml) was added to the tube which was carefully inverted 5 times to wash the DNA. A new centrifugation was performed (10,000 rpm for 5 min). Supernatant was discarded and the pellet was air-dried for 15 min at fume hood. After that, DNA was resuspended in 50 μl of sterile Milli-Q water and was placed in a dry bath at 60 °C for 15 min to complete DNA solubilization. Except the maceration and isopropanol addition, all steps were performed at room temperature.

### Bud length and leaf measurements

For bud length measurements, plants were cultivated in 300 ml pots at the greenhouse. Each axillary bud in the primary stem was measured. Mean bud length in each plant was obtained by dividing the total branch length by the number of branches. Number of leaves on the primary shoot to the first inflorescence were counted and the total leaf area was measured considering all leaves of each plant. For leaf area, leaves were digitalized using 300 DPI in a HP Scanner (Hewlett Packard, Palo Alto, CA, USA). Images were processed and analyzed using the ImageJ (http://imagej.nih.gov/ij/). All measurements were taken at anthesis.

### Seedling and meristem frequency

Seedling emergence frequency was measured between 4 to 7 days after sowing (DAS). The number of emerged seedlings in each day was divided by the total number of seedlings (41 for MT and 42 for CKX2-OE#2) to obtain the emergence rate. The SAM maturation was measured along the time and was estimated by the frequency of each vegetative, transition and inflorescence meristem frequency. These data were obtained by dividing the number of meristems in each stage by the total number of meristems evaluated in each DAE. Data are showed in percent.

### CK response assays

To measure the cytokinins response in roots, in vitro grown plants were treated with the cytokinins isopentenyladenine (iP) or thidiazuron (TDZ). Seeds were surface-sterilized and germinated in half strength MS medium, B5 vitamins, 15 g/l sucrose and 7 g/l agar. Emerged seedlings were transferred to Petri dishes containing MS medium, B5 vitamins, 30 g/l sucrose, 7 g/l agar and increasing concentrations of iP or TDZ. Seedlings were grown vertically oriented on the surface of this medium growth room under 16 h photoperiod at 25 ± 1 °C for 7 d. The total root length (sum of primary root, lateral and adventitious roots) was measured using a graduated ruler.

For cytokinin response in axillary bud induction, BAP in lanolin was supplied to the leaf axils of vegetative plants, with two pairs of expanding leaves. Plants were grown in the greenhouse as above described. BAP was dissolved in dimethylsulfoxide (DMSO) to make 100 mM stock and then mixed to a pre-warmed lanolin (50 °C) to final concentration of 10 mM. For control treatment, a mock solution was prepared with the identical volume of pre-warmed lanolin and DMSO. Lanolin paste (BAP or mock) was immediately applied to each leaf axil using a pipette tip. Bud length was measured 13 d after BAP or mock treatments.

### CK quantification

Plants for t-Zeatin and iP (N^6^-(∆^2^isopentenyl) adenine) analysis were grown on soil. Shoots and roots were collected at the same developmental stage of the plant (i.e., when six leaves were formed). The samples were flash frozen using liquid nitrogen and kept at − 80 °C. Then the samples were dried in a Labconco Freezone 4.5 freeze-dryer (Labconco Corporation, Kansas, Missouri, USA) land ground to a fine powder. The extraction and analysis of hormones were conducted using the same procedures reported by Albacete et al. (2008). Five and three independently pooled samples of lyophilized powder were analyzed. Fifty mg of shoots or 20 mg of roots were respectively dropped in 1080 and 430 μl of cold (4 °C) extraction buffer obtained from mixing methanol/water (80/20, v/v). The tubes were mixed three times at intervals of 10 min. Solids were separated by centrifugation (13,000 g, 15 min) and supernatant was saved and maintained at 4 °C. The obtained solids were submitted to same extraction procedure one more time.

After the second centrifugation, supernatants were pooled and passed through Sep-Pak Plus †C18 cartridge (SepPak Plus, Waters, USA) and the liquid was dried at 40 °C under vacuum. The residue was dissolved in 1 ml methanol/water (20/80, v/v) solution using an ultrasonic bath (25 °C, 10 min). Afterwards the extracts were centrifuged twice (13,000 g, 15 min, 4 °C. Ten μl of each extract were injected to U-HPLC-MS system consisting of an Accela Series U-HPLC (Thermo Fisher Scientific, Waltham, MA, USA) coupled to an Exactive Mass Spectrometer (Thermo Fisher Scientific, Waltham) with a heated electrospray ionization (HESI) interface. Mass spectra were obtained using the Xcalibur software v, 2.2 (ThermoFisher Scientific). For quantification of plant hormones, calibration curves were obtained for each analyzed component (1, 10, 50, and 100 μg/l) and corrected for 10 μg/l deuterated internal standards. Recovery rates ranged between 92 and 95%.

### Grafting

For in vitro micrografting, seedlings were germinated in the same conditions established for genetic transformation. Cultures were incubated in the dark for 4 d at 25 ± 1 °C. Just-emerged seedlings were transferred to Petri dishes (3 per dish) containing MS supplemented with B5 vitamins, 30 g/l sucrose and 7 g/l agar in the horizontal position. Dishes were positioned at approximately 110 degrees of inclination to allow roots penetrate in the medium. Seedlings were micrografted using a transversal cut done in the hypocotyl after 5 to 9 days using a scalpel in MT and CKX2-OE#2 genotypes, with the following combinations between scion and rootstock: MT/MT, CKX2-OE#2/CKX2-OE#2, MT/CKX2-OE#2 and CKX2-OE#2/MT. Plates with the micrografted seedlings were incubated in growth room under 16 h photoperiod at 25 ± 1 °C for 10 d. Plantlets were hardened in the greenhouse and evaluated at anthesis.

### RNA extraction and cDNA synthesis

For gene expression analysis, plants were growth in the greenhouse. Axillary buds (0.5 to 1.0 cm) were harvested from the 3th and 4th node of vegetative plants and hypocotyls were harvested from 2-week-old seedlings. These materials were immediately frozen in liquid nitrogen. Total RNA was extracted from approximately 100 mg fresh weight following the protocol of the manufacturer Trizol®Reagent (Ambion, Life Technologies, Carlsbad, CA, USA). RNA integrity was analyzed on a 1% w/v agarose gel. The RNA concentration was estimated before and after treatment with DNase (RQ1 RNase-free DNase, Promega, Madison, WI, EUA) using a Nanodrop One spectrophotometer (http://www.nanodrop.com/). DNase I-treated RNA (2 μg) from three biological replicates was reverse-transcribed to generate first-strand cDNA according to manufacturer’s instructions RevertAid RT Kit (Thermo Fisher Scientific). Each replicate consisted of a pool of five axillary buds from two or three plants.

### Reverse transcription-quantitative PCR

Quantitative PCR was performed using Platinum SYBR Green qPCR SuperMix UDG (Invitrogen) and the Rotor-Gene 3000 (Corbett Research, Sidney, Australia), using 1 μL of each diluted sample (40 folds) as a template in a 10-μL reaction. All qPCRs were performed with an annealing temperature of 60 °C. Two technical replicates were performed on each of the three independently biological samples including the template-free reactions as negative controls. Relative expression was estimated using *ACTIN* (Solyc04g011500) and *UBUQUITIN* (Solyc04g081490) as gene references. *ACTIN* was used as a normalizer to calculate the fold-changes for each gene based on the cycle threshold (CT) using the eq. 2^-ΔΔCT^ (Livak and Schmittgen, 2001) Primer sequences used are shown in Table S2.

### Auxin transport and response analysis

Hypocotyl segments of 2-week-old seedlings were used for basipetal polar auxin transport (PAT) assay as described by Al-Hammadi et al. (2003), with some modifications. Hypocotyl segments (~ 10 mm in length) were excised and incubated in 5 mM phosphate buffer (pH 5.8) containing 1 μM IAA for 2 h at 25 °C ± 2 °C on a rotary shaker (200 rpm) under dark conditions. Agar donor blocks (1% [w/v] agar in 5 mM phosphate buffer [pH 5.8]) containing 1 μM IAA and 100 nM [^3^H] IAA were placed in contact with the apical end of hypocotyl sections, and an agar receiver block (1% [w/v] agar in water) was placed on the basal end of each section. The system was incubated for 4 h in a humid chamber at 25 °C ± 2 °C. After that, the receiver blocks were removed and incubated overnight in a 3-mL scintillation cocktail in the dark and under shaking (100 rpm) at 28 °C ± 2 °C. Analysis was performed in a scintillation counter (Ultima Gold; PerkinElmer, Waltham, Massachusetts, EUA). As a negative control, hypocotyl segments were sandwiched for 30 min between NPA-containing blocks (1% [w/v] agar in water containing 20 mM NPA) prior to the auxin transport assays. ^3^H disintegrations per minute (dpm) values in the receiver block were converted to fmol of auxin transported as described by Lewis and Muday (2009).

For decapitation experiment, the shoot apex of plants with two pairs of expanding leaves was excised. Plants were grown in the greenhouse as described above. IAA was dissolved in DMSO to make 1 mM stock and then mixed to a pre-warmed lanolin (50 °C) to final concentration of 10 μM. For control treatment, a mock solution was prepared with the identical volume of pre-warmed lanolin and DMSO. Lanolin paste (IAA or mock treatment) was immediately applied to the cut stump using a pipette tip. Bud length was measured 10 days after IAA or mock treatments.

### Histochemical assays

Transgenic MT and *CKX2-OE* double transgenic DR5::GUS plants were incubated overnight at 37 °C in GUS staining solution [80 mM sodium phosphate buffer, pH 7.0; 0.4 mM potassium ferrocyanide; 8 mM EDTA; 0.05% Triton X-100; 0.8 mg/mL 5-bromo-4-chloro-3-indolyl-b-D-glucuronide (X-Gluc); 20% methanol]. After GUS staining, samples were washed in 70% (w/v) ethanol to remove chlorophyll. Samples were then photographed using a Leica S8AP0 microscope coupled to a Leica DFC295 camera.

### Histological analysis

Samples were collected from in vitro-cultured seedlings and were fixed in Karnovsky solution (Karnovsky, 1965) for 24 h. After that, they were dehydrated in an increasing ethanol series (10–100%) and subsequently infiltrated with synthetic resin using a Historesin embedding kit (Leica, www.leica-microsystems.com), according to the manufacturer’s instructions. Tissue sections (5 μm) were obtained using a rotary microtome (Leica) and stained with toluidine blue 0.05% in a phosphate buffer and citric acid pH 4.0 (Sakai, 1973). Permanent slides were mounted with synthetic resin (EntellanR, Merck).

### Statistical analysis

Statistical analyses were performed using SAS software. Data were analyzed by using one-way ANOVA and two-tailed Student’s t-test and Mann Whitney test (*P < 0.05, **P < 0.01, ***P < 0.001) and Tukey test (*P* < 0.05).

### Supplementary Information

**Additional file 1: Table S1.** Vegetative development of *CKX2*-overexpressing tomato plants. ^1^Number of days taken from sowing to open the first flower in the primary stem. ^2^Number of leaves on the primary shoot for the first inflorescence. Total leaf area was measured considering all leaves of each plant. Data are means ± SE (*n* = 10 plants). Different letters indicate significant differences among genotypes (Student’s *t*-test *P* < 0.01). **Table S2.** Gene-specific primers used for qRT-PCR analysis. ^1^Locus according to the Sol Genomics Network database (http://solgenomics.net/). ^2^Fwd: forward, Rev.: reverse

**Additional file 2: Figure S1.** Compound leaf development is altered in CKX2-overexpressing tomato plants. Fully-expanded fifth leaf from each genotype at 45 DAE. Notice the smaller leaflets in the transgenic lines with smooth leaf margins. **Figure S2.** Tomato CKX2-overexpressing plants delay initial development and the transition to reproductive development. **A**. Seedlings considered as emerged from that stage; bar = 1 cm. **B**. Seedling emergence frequency between 4 to 7 days after sowing (DAS). **C to H**. Representative images of meristem maturation of the primary shoot meristem of MT (C, E and G) and CKX2-OE#2 (D, F and H) in the vegetative (C and D), transition (E and F) and reproductive (G and H) stages; bar = 200 μm. **I and J**. Vegetative to reproductive primary meristem transition evaluated by the seedling frequency in each developmental stage in MT (I) and CKX2-OE#2 (J), from the 1st to 55th DAE (days after emergence). **K and L**. Representative bud axil initiation in the 2nd leaf axil of MT (K) and CKX2-OE#2 (L); bar = 400 μm. **M-P**. Schematic representation of axillary bud formation in leaf axils at 13 and 16 DAE in MT (M and N) and in CKX2-OE#2 (O and P). **Figure S3.** Shoot phenotype of CKX2-overexpressing tomato plants. The shoot development was registered in MT and CKX2-OE tomato plants at 14 DAE (**A**), 28 DAE (**B**) and 32 DAE (**C**). DAE, days after emergence. **Figure S4.** Root development pattern is altered in tomato plants overexpressing CKX2. **A**. Comparison of root growth of MT and CKX2-OE#2 seedlings at 4, 8, 12 and 16 days after emergence (DAE). **B**. Primary root length. **C**. Total root length. **D**. Number of lateral roots. **E**. Number of adventitious roots. Data are means ± se (*n* = 10). B-D: asterisks indicate significant differences between genotypes within a treatment (B and C: Student’s *t*-test; D: Mann Whitney test; **P < 0.01, ***P < 0.001; E:letters indicate significant differences among treatments within the same genotype (Tukey test; *P* < 0.05). **Figure S5.** Tomato CKX2-overexpressing plants alter SAM development, vessels organization and root development. Apical shoot (**A** and **B**), hypocotyl (**C** and **D**) and primary root (**E** and **F**) were collected from MT (A, C and E) and CKX2-OE#2 (B, D and F) seedlings at 7 DAE. Apical shoots were longitudinally sectioned while hypocotyls and roots were cross-sectioned. Bar = 100 μm. **Figure S6.** Reduced *ARR5::GUS* and *DR5::GUS* expression in the CKX2-overexpressing tomato plants. The *ARR5::GUS* (A and B) and DR5::GUS (C and D) expression was evaluated in apical shoots of MT (A and C) and CKX2-OE#2 (B and D) seedlings. Bars = 500 μm (A, C and D) and 200 μm (B). **Figure S7.** Bud length of tomato plants overexpressing *CKX2* upon application of BAP. Shoot phenotype (**A**) and mean bud length (**B**) of BAP-treated and non-treated CKX2-OE#2 compared to MT plants. BAP (10 mM) or mock (0 mM BAP) was applied in the leaf axil of MT and CKX2-OE#2 plants at the early vegetative (i.e., 20 DAE for MT and 38 DAE for CKX2-OE#2). The length of the bud in each axil were measured 13 days after treatment. Data are means ± se (*n* = 12). Asterisks indicate significant differences between treatments within the same genotype (Mann Whitney test; **P < 0.01, ***P < 0.001). **Figure S8.** Increased seed weight of CKX2-overexpressing tomato plants. The weight of one seed was calculated from the weight of pools of 100 seeds. Data are means ± SE (*n* = 12). Asterisk indicate significant difference between genotypes (Student’s t-test; ***P < 0.001).

## Data Availability

The data supporting the findings of this study and the tomato genotypes used are available from the corresponding author, (Lázaro E. P. Peres), upon request.

## Abbreviations

OE: Overexpressing
LC-MS: Liquid Chromatography Mass Spectrometry
TCP: TCP protein
AS: Antisense
AMs: Axillary Meristems
PAT: Polar Auxin Transport
CK: Cytokinin
iPMP: isopentenyadenosine-5′-monophosphate
MT: Micro-Tom
DAS: Days After Sowing
SAM: Shoot Apical Meristem
DAG: Days After Germination
TDZ: Thidiazuron
iP: isopentyladenine
t-Zeatin: trans-Zeatin
GUS: beta-glucuronidase
BAP: Benzylaminopurine
SL: Strigolactone
BC: Backcross
PCR: Polymerase Chain Reaction
MS: Murashige & Skoog Medium
DMSO: Dimethylsulfoxide
U-HPLC-MS: Ultra-high performance liquid chromatography-tandem mass spectrometry
CT: Cycle Threshold
IAA: Indole-3-acetic acid
NPA: N-1-Naphthylphthalamic Acid
EDTA: Ethylenediaminetetraacetic Acid
