# Supplementary material for: Increased branching independent of strigolactone in cytokinin oxidase 2-overexpressing tomato is mediated by reduced auxin transport
Source: Mol Hortic. 2022 May 3;2:12. doi: 10.1186/s43897-022-00032-1 (PMC10514996; doi:10.1186/s43897-022-00032-1)
Supplement: Supplementary file 1 — Additional file 1: Table S1. Vegetative development of CKX2-overexpressing tomato plants. 1Number of days taken from sowing to open the first flower in the primary stem. 2Number of leaves on the primary shoot for the first inflorescence. Total leaf area was measured considering all leaves of each plant. Data are means ± SE (n = 10 plants). Different letters indicate significant differences among genotypes (Student’s t-test P < 0.01). Table S2. Gene-specific primers used for qRT-PCR analysis. 1Locus according to the Sol Genomics Network database (http://solgenomics.net/). 2Fwd: forward, Rev.: reverse [file 43897_2022_32_MOESM1_ESM.docx]

**Supplemental Information**

**Table S1.** Vegetative development of *CKX2*-overexpressing tomato plants.

|  |  |  | CKX2-OE |  |
| --- | --- | --- | --- | --- |
|  | MT | #1 | #2 | #3 |
| Time to anthesis^1^ | 41.10 ± 0.94**^d^** | 65.5 ± 2.82**^a^** | 57.6 ± 1.04**^b^** | 51.1 ± 0.79**^c^** |
| No. of leaves on the primary shoot^2^ | 6.70 ± 0.30**^c^** | 8.10 ± 0.27**^b^** | 9.50 ± 0.16**^a^** | 7.50 ± 0.16**^b^** |
| Total leaf area per plant (cm^2^) | 306.25 ± 15.26**^a^** | 163.76 ± 4.73**^c^** | 151.04 ± 7.93**^c^** | 221.31 ± 15.85**^b^** |

^1^Number of days taken from sowing to open the first flower in the primary stem. ^2^Number of leaves on the primary shoot for the first inflorescence. Total leaf area was measured considering all leaves of each plant. Data are means ± SE (n = 10 plants). Different letters indicate significant differences among genotypes (Student’s *t*-test P < 0.01).

**Table S2**. Gene-specific primers used for qRT-PCR analysis.

| **Gene** | **Locus ID^1^** | **Primers sequence^2^** |
| --- | --- | --- |
| *Actin* | Solyc03g078400 | Fwd 5’-GGTCCCTCTATTGTCCACAG-3’  Rev 3’-TGCATCTCTGGTCCAGTAGGA-5’ |
| *Ubiquitin* | Solyc04g081490 | Fwd 5’-AACCTCCATTCAGGAGATGTTT-3’  Rev 3’-TCTGCTGTAGCATCCTGGTATT-5’ |
| *AtCKX2* | AT2G19500 | Fwd 5’-CTCGCGACAGCTAAACCCAG-3’  Rev 3’-ATCCGAAACCATTTGGCCCG-5’ |
| *SlIPT4* | Solyc09g064910 | Fwd 5’-ACACCAAAGGTATTCGTAAAGCCA-3’  Rev 3’-CGCTTCCTCTAGCATCCTCTCA -5’ |
| *SlIPT5* | Solyc11g066960 | Fwd 5’-GACTGTAGAGAACTGGGGTCAA-3’  Rev 3’-TCCAAAGTGGTCAGAGATGC-5’ |
| *SlLOG1* | Solyc11g069570 | Fwd 5’-TGCACCAGAGGAAAGCAGAGA-3’  Rev 3’-TTCCGAGTTGAGACCACGCT-5’ |
| *SlLOG4* | Solyc01g005680 | Fwd 5’-GCTGGCTTGATCCTGTCATCT-3’  Rev 3’-CTCTCTTGCTACTCAAAACCAAACC-5’ |
| *SlLOG5* | Solyc08g062820 | Fwd 5’-CATGTTGCTCCCCATGAAA-3’  Rev 3’-TGGAGACTGCTCCTTTGGAT-5’ |
| *SlTRR3* | Solyc03g113720 | Fwd 5’-GCAGTGGAAAGTGGAACGAG-3’  Rev 3’-CCAGTCATCCCAGGCATAGAA-5’ |
| *SlBRC1a* | Solyc03g119770 | Fwd 5’-GAATTCGGGTTCATATTGCAT-3’  Rev 3’-AAAGATGTACCCTTCGAGCAA-5’ |
| *SlBRC1b* | Solyc06g069240 | Fwd 5’-GACAAAAACAAGGAAACCGCTCA-3’  Rev 3’-CCCTGGCTCTTGCTTGATTCC-5’ |
| *SlPIN1* | Solyc03g118740 | Fwd 5’-ACAATGGCAACAAAGCACACAA -3’  Rev 3’-GTCCTCCAAAAACATCAGAAACAGG-5’ |
| *SlPIN4* | Solyc05g008060 | Fwd 5’-GGGGAATTTGGTGGTGAGGATTT-3’  Rev 3’-AGCCGGAGGCATCTGTTTTC-5’ |
| *SlPIN7* | Solyc10g080880 | Fwd 5’-CTCTCCGATGCTGGTCTTGG-3’  Rev 3’-ACTGCTGGACCGCTGATGAA-5’ |

^1^Locus according to the Sol Genomics Network database (http://solgenomics.net/).

^2^Fwd: forward, Rev: reverse
