# Supplementary material for: Increased branching independent of strigolactone in cytokinin oxidase 2-overexpressing tomato is mediated by reduced auxin transport
Source: Mol Hortic. 2022 May 3;2:12. doi: 10.1186/s43897-022-00032-1 (PMC10514996; doi:10.1186/s43897-022-00032-1)
Supplement: Supplementary file 2 — Additional file 2: Figure S1. Compound leaf development is altered in CKX2-overexpressing tomato plants. Fully-expanded fifth leaf from each genotype at 45 DAE. Notice the smaller leaflets in the transgenic lines with smooth leaf margins. Figure S2. Tomato CKX2-overexpressing plants delay initial development and the transition to reproductive development. A. Seedlings considered as emerged from that stage; bar = 1 cm. B. Seedling emergence frequency between 4 to 7 days after sowing (DAS). C to H. Representative images of meristem maturation of the primary shoot meristem of MT (C, E and G) and CKX2-OE#2 (D, F and H) in the vegetative (C and D), transition (E and F) and reproductive (G and H) stages; bar = 200 μm. I and J. Vegetative to reproductive primary meristem transition evaluated by the seedling frequency in each developmental stage in MT (I) and CKX2-OE#2 (J), from the 1st to 55th DAE (days after emergence). K and L. Representative bud axil initiation in the 2nd leaf axil of MT (K) and CKX2-OE#2 (L); bar = 400 μm. M-P. Schematic representation of axillary bud formation in leaf axils at 13 and 16 DAE in MT (M and N) and in CKX2-OE#2 (O and P). Figure S3. Shoot phenotype of CKX2-overexpressing tomato plants. The shoot development was registered in MT and CKX2-OE tomato plants at 14 DAE (A), 28 DAE (B) and 32 DAE (C). DAE, days after emergence. Figure S4. Root development pattern is altered in tomato plants overexpressing CKX2. A. Comparison of root growth of MT and CKX2-OE#2 seedlings at 4, 8, 12 and 16 days after emergence (DAE). B. Primary root length. C. Total root length. D. Number of lateral roots. E. Number of adventitious roots. Data are means ± se (n = 10). B-D: asterisks indicate significant differences between genotypes within a treatment (B and C: Student’s t-test; D: Mann Whitney test; **P < 0.01, ***P < 0.001; E:letters indicate significant differences among treatments within the same genotype (Tukey test; P < 0.05). Figure S5. Tomato CKX2-overexpre [file 43897_2022_32_MOESM2_ESM.pdf]

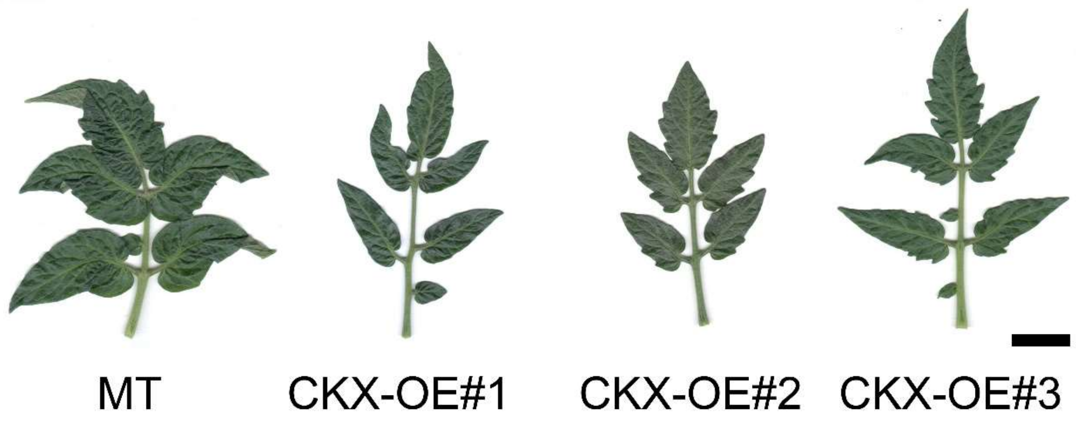


**Figure S1. Compound leaf development is altered in CKX2-overexpressing tomato plants**. Fully-expanded fifth leaf from each genotype at 45 DAE. Notice the smaller leaflets in the transgenic lines with smooth leaf margins.

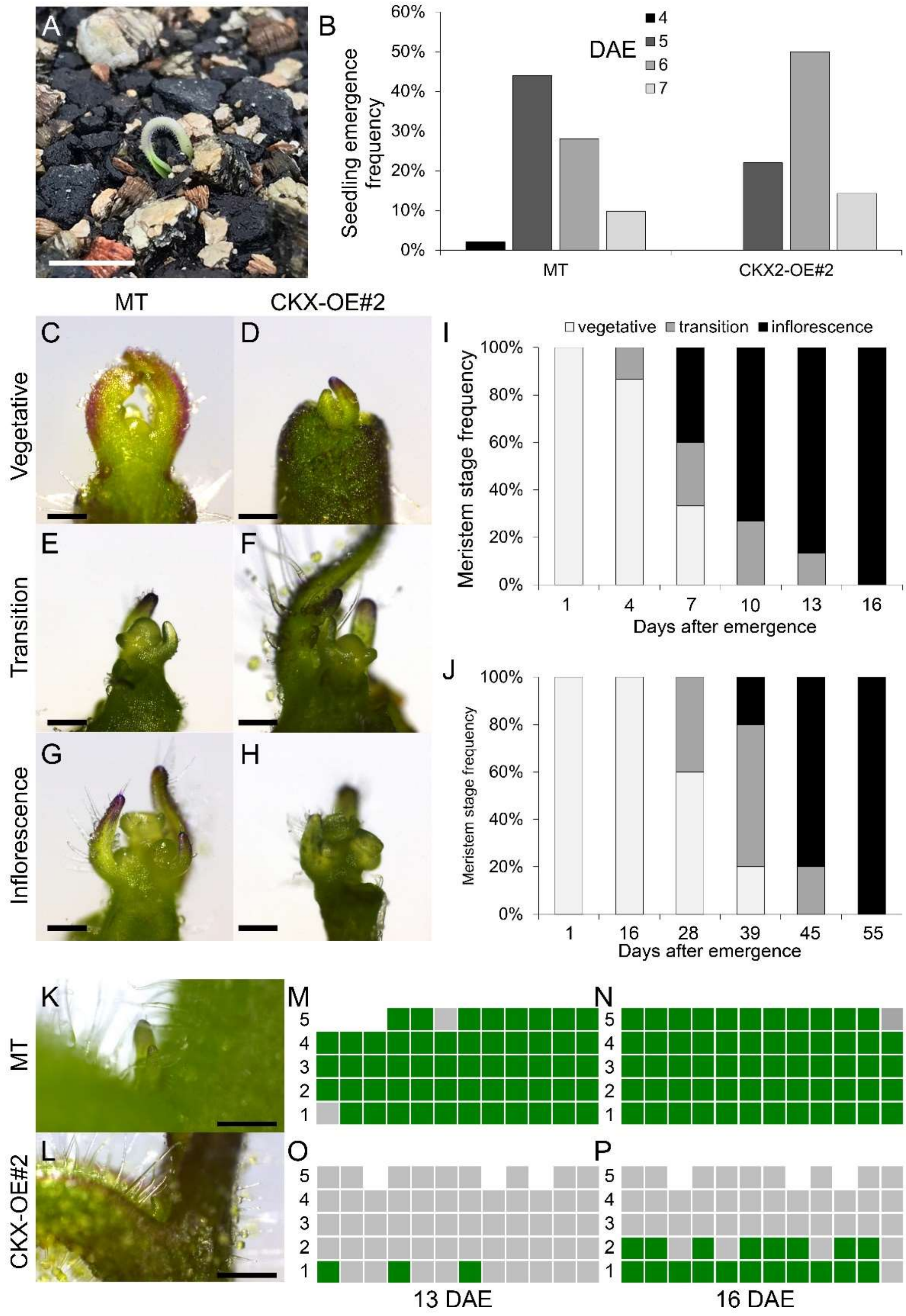


**Figure S2. Tomato CKX2-overexpressing plants delay initial development and the transition to reproductive development**. **A**. Seedlings considered as emerged from that stage; bar = 1cm. **B**. Seedling emergence frequency between 4 to 7 days after sowing (DAS). **C to H**. Representative images of meristem maturation of the primary shoot meristem of MT (C, E and

G) and CKX2-OE#2 (D, F and H) in the vegetative (C and D), transition (E and F) and reproductive (G and H) stages; bar = 200 µm. **I and J**. Vegetative to reproductive primary meristem transition evaluated by the seedling frequency in each developmental stage in MT (I) and CKX2-OE#2 (J), from the 1st to 55th DAE (days after emergence). **K and L**. Representative bud axil initiation in the 2nd leaf axil of MT (K) and CKX2-OE#2 (L); bar = 400 µm. **M to P**. Schematic representation of axillary bud formation in leaf axils at 13 and 16 DAE in MT (M and N) and in CKX2-OE#2 (O and P).

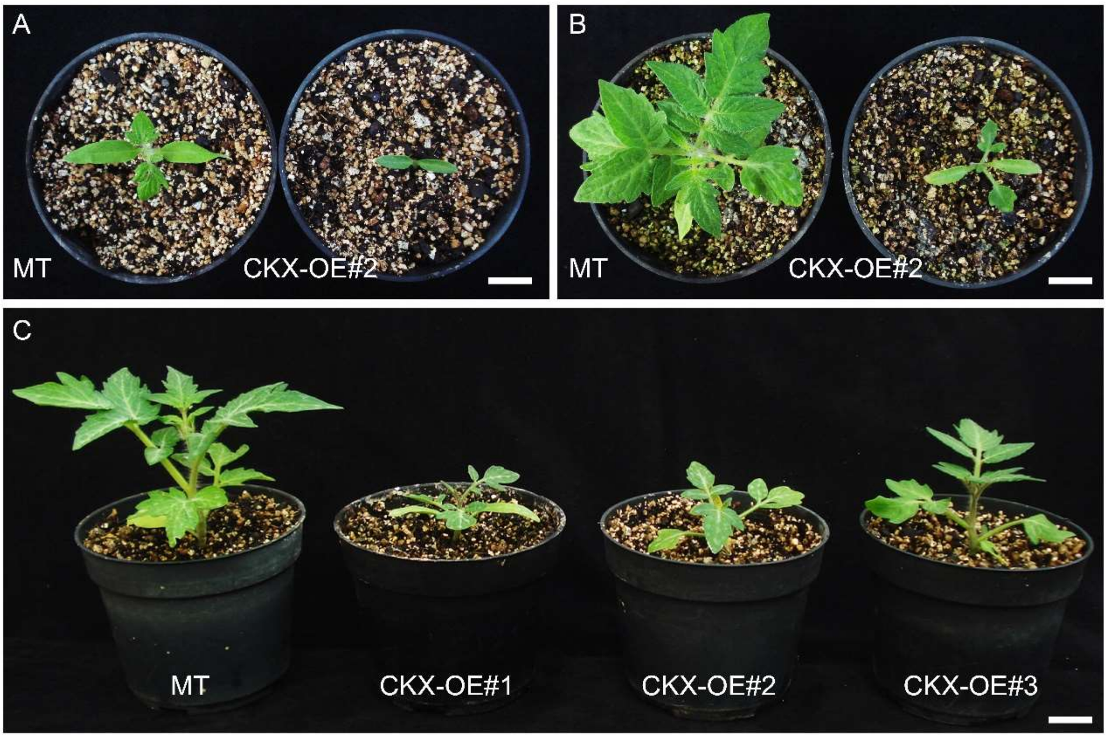


**Figure S3. Shoot phenotype of CKX2-overexpressing tomato plants.** The shoot development was registered in MT and CKX2-OE tomato plants at 14 DAE (**A**), 28 DAE (**B**) and 32 DAE (**C**). DAE, days after emergence.

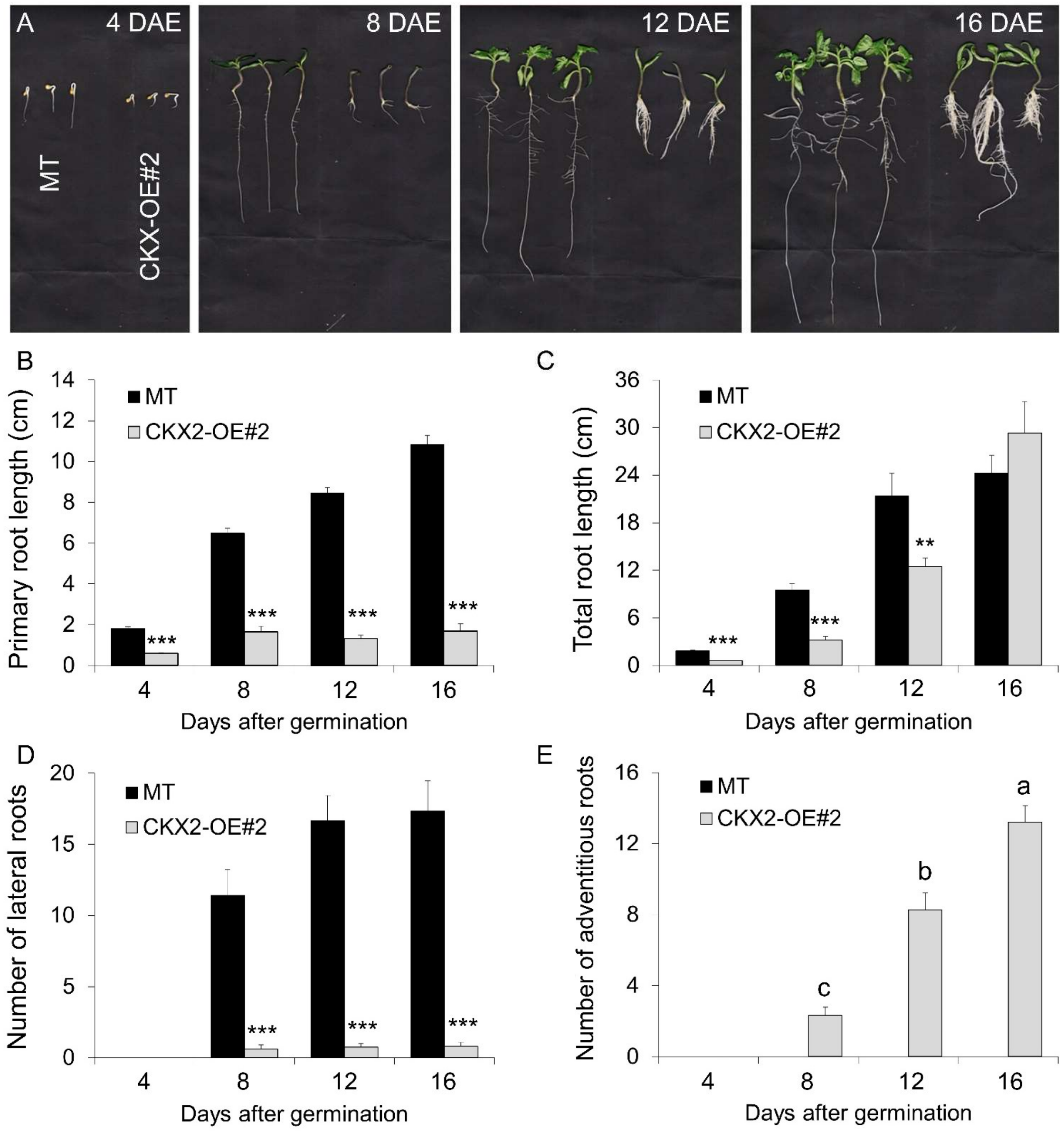


**Figure S4**. **Root development pattern is altered in tomato plants overexpressing *CKX2***. **A**. Comparison of root growth of MT and CKX2-OE#2 seedlings at 4, 8, 12 and 16 days after emergence (DAE). **B**. Primary root length. **C**. Total root length. **D**. Number of lateral roots. **E**. Number of adventitious roots. Data are means ± SE (n = 10). B-D: asterisks indicate significant differences between genotypes within a treatment (B and C: Student's *t*-test; D: Mann Whitney test; **P < 0.01, ***P < 0.001; E: letters indicate significant differences among treatments within the same genotype (Tukey test; P < 0.05).

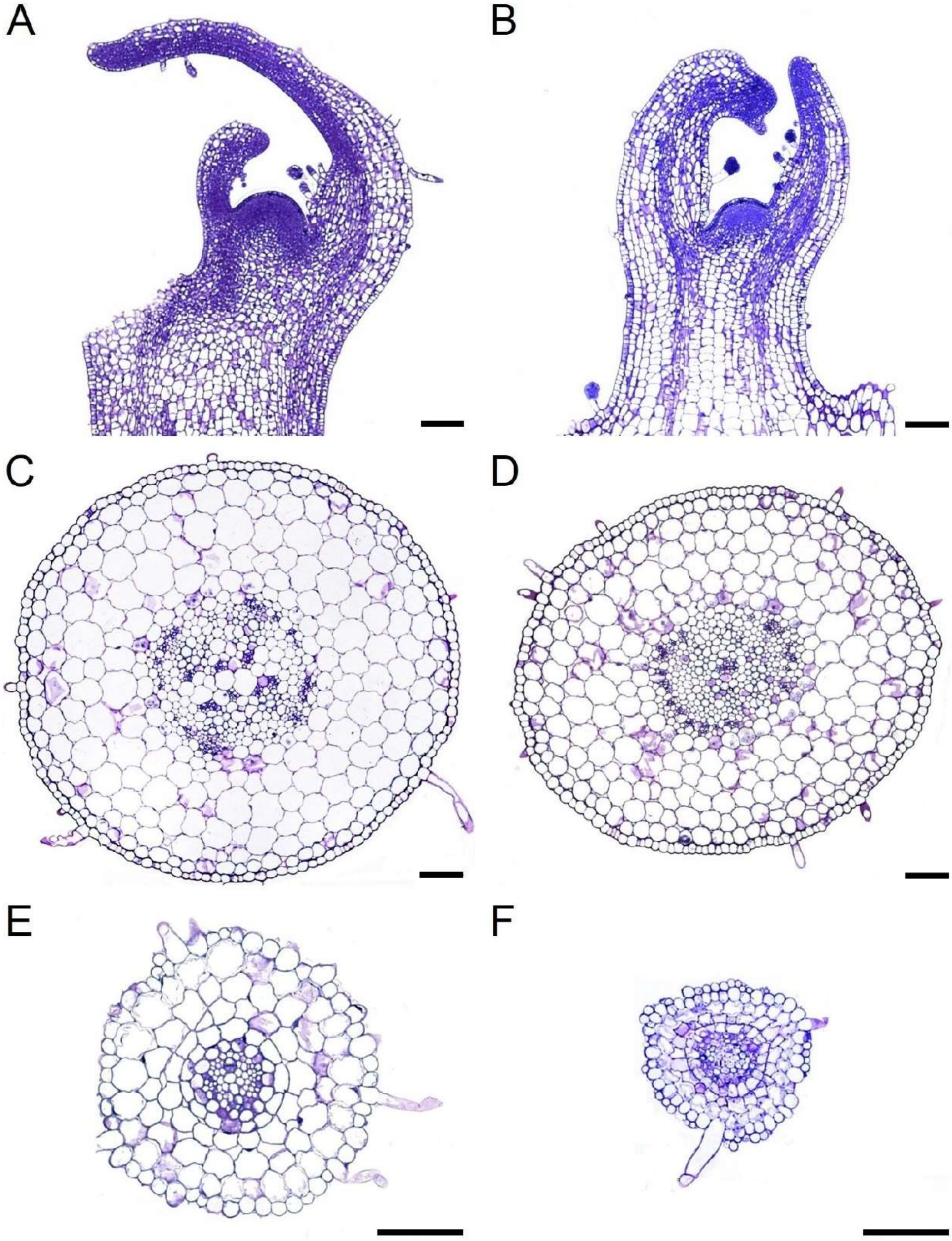


**Figure S5**. **Tomato CKX2-overexpressing plants alter SAM development, vessels organization and root development**. Apical shoot (**A** and **B**), hypocotyl (**C** and **D**) and primary root (**E** and **F**) were collected from MT (A, C and E) and CKX2-OE#2 (B, D and F) seedlings at

7 DAE. Apical shoots were longitudinally sectioned while hypocotyls and roots were cross-sectioned. Bar = 100 µm.

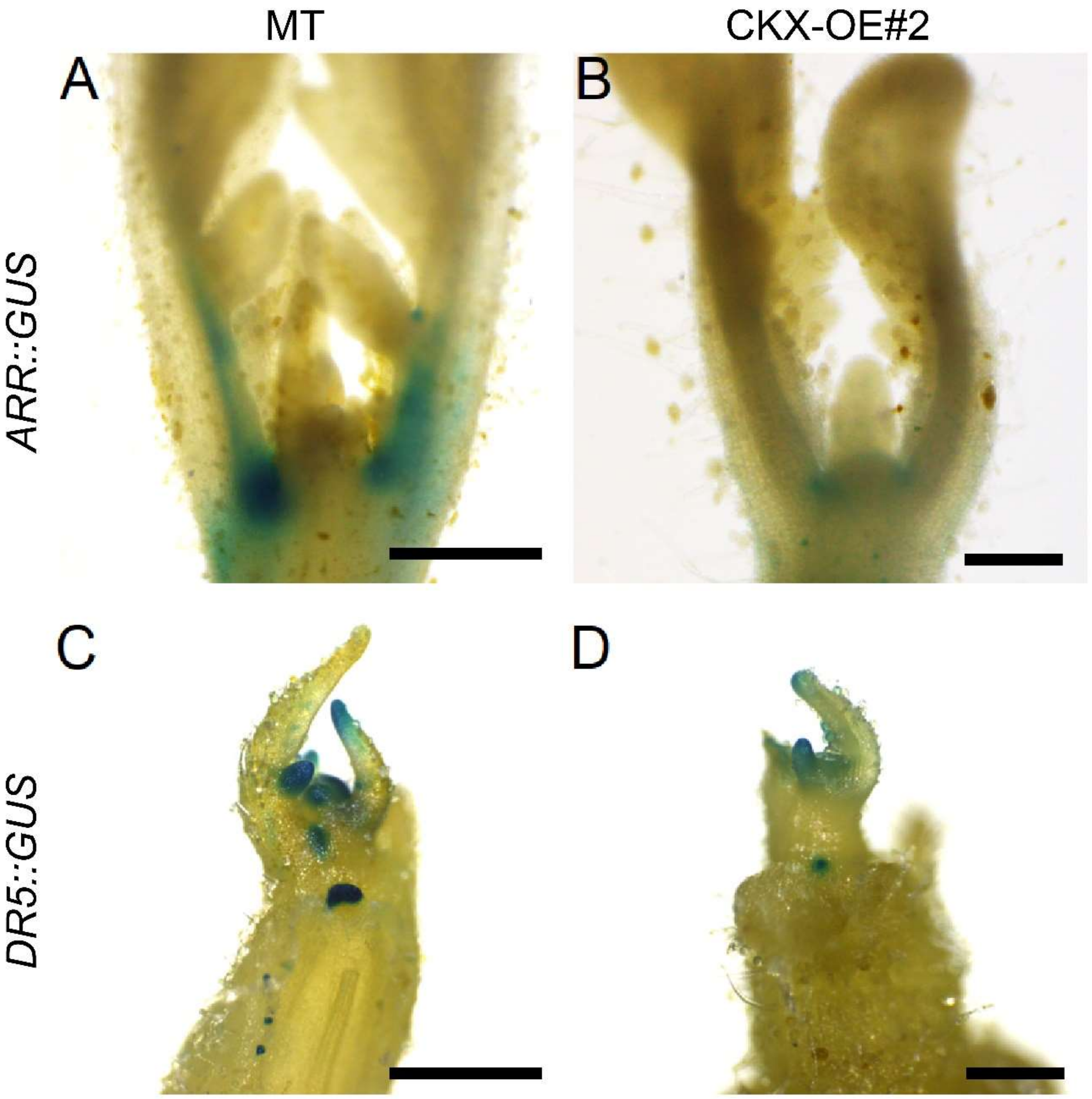


**Figure S6. Reduced *ARR5::GUS* and *DR5::GUS* expression in the CKX2-overexpressing tomato plants.** The *ARR5::GUS* (A and B) and DR5::GUS (C and D) expression was evaluated in apical shoots of MT (A and C) and CKX2-OE#2 (B and D) seedlings. Bars = 500 µm (A, C and D) and 200 µm (B).

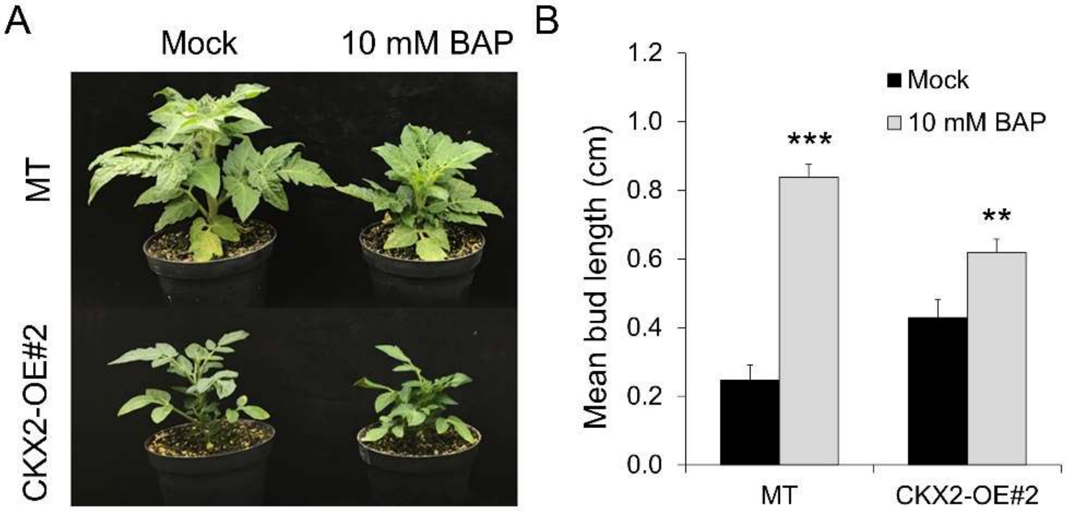


**Figure S7. Bud length of tomato plants overexpressing *CKX2* upon application of BAP.** Shoot phenotype (**A**) and mean bud length (**B**) of BAP-treated and non-treated CKX2-OE#2 compared to MT plants. BAP (10 mM) or mock (0 mM BAP) was applied in the leaf axil of MT and CKX2-OE#2 plants at the early vegetative (i.e., 20 DAE for MT and 38 DAE for CKX2-OE#2). The length of the bud in each axil were measured 13 days after treatment. Data are means ± SE (n =12). Asterisks indicate significant differences between treatments within the same genotype (Mann Whitney test; **P < 0.01, ***P < 0.001).

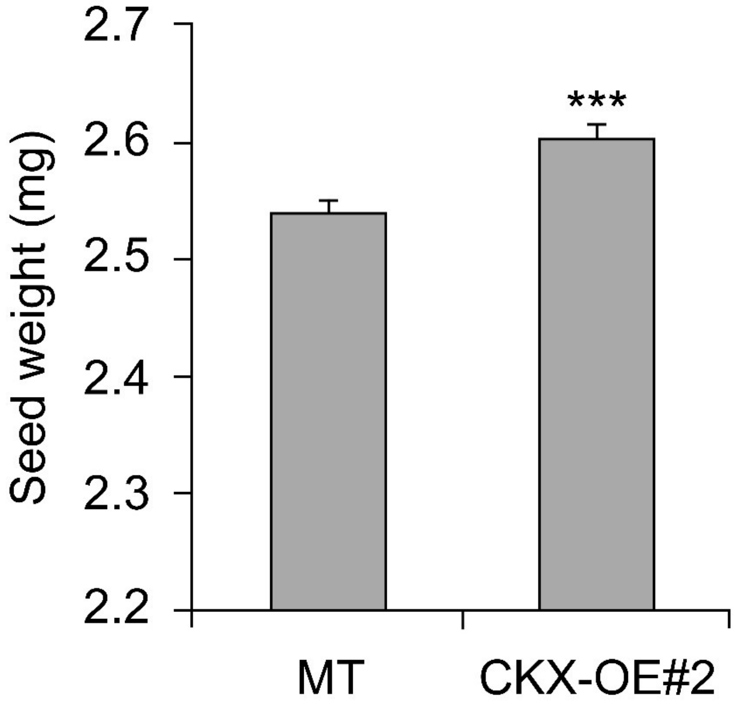


**Figure S8. Increased seed weight of CKX2-overexpressing tomato plants.** The weight of one seed was calculated from the weight of pools of 100 seeds. Data are means ± SE (n =12). Asterisk indicate significant difference between genotypes (Student's t-test; ***P < 0.001).
